# Enantiomerization of Axially Chiral Biphenyls: Polarizable MD Simulations in Water and Butylmethylether

**DOI:** 10.3390/ijms21176222

**Published:** 2020-08-28

**Authors:** Veronika Zeindlhofer, Phillip Hudson, Ádám Márk Pálvölgyi, Matthias Welsch, Mazin Almarashi, H. Lee Woodcock, Bernard Brooks, Katharina Bica-Schröder, Christian Schröder

**Affiliations:** 1 Department of Computational Biological Chemistry, University of Vienna, Währingerstraße 17, 1090 Vienna, Austria; veronika.zeindlhofer@univie.ac.at (V.Z.); m.welsch@chello.at (M.W.); a01619644@unet.univie.ac.at (M.A.); 2 Laboratory of Computational Biology, National Heart, Lung, and Blood Institute (NHLBI), National Institutes of Health (NIH), Bethesda, MD 20892, USA; phillip.hudson@nih.gov (P.H.); brb@mail.nih.gov (B.B.); 3 Department of Chemistry, University of South Florida, Tampa, FL 33620, USA; hlw@mail.usf.edu; 4 Institute of Applied Synthetic Chemistry, TU Wien, Getreidemarkt 9/163, 1060 Vienna, Austria; adam.palvoelgyi@tuwien.ac.at (Á.M.P.); katharina.schroeder@tuwien.ac.at (K.B.-S.)

**Keywords:** chiral ionic liquids, biphenyl, chirality transfer, molecular dynamics simulations

## Abstract

In this study, we investigate the influence of chiral and achiral cations on the enantiomerization of biphenylic anions in *n*-butylmethylether and water. In addition to the impact of the cations and solvent molecules on the free energy profile of rotation, we also explore if chirality transfer between a chiral cation and the biphenylic anion is possible, i.e., if pairing with a chiral cation can energetically favour one conformer of the anion via diastereomeric complex formation. The quantum-mechanical calculations are accompanied by polarizable MD simulations using umbrella sampling to study the impact of solvents of different polarity in more detail. We also discuss how accurate polarizable force fields for biphenylic anions can be constructed from quantum-mechanical reference data.

## 1. Introduction

Over the last 20 years, chiral ionic liquids (CILs) have been in the focus of a rapidly growing field of research [1,2,3,4]. Although the first synthesis of a CIL was reported in 1997 [5], it took seven more years until the first successful chirality transfer by a CIL in an asymmetric Baylis–Hillman reaction was realized by Pégot et al. in 2004 [6]. However, since then the reports on successful applications of CILs in analytics [7,8,9,10] and asymmetric synthesis have been increasing steadily. In asymmetric synthesis, CILs can be used either as reaction solvent [11,12,13] or incorporated into the catalytic system, for example, as chiral ligand [3,14,15,16] or chiral organocatalyst [17,18].

Although chirality transfer via ion pairing in asymmetric synthesis (counterion directed catalysis) has been successfully realized in many high-impact studies [19,20,21,22,23], few examples that utilize ion aggregation in CILs for asymmetric synthesis exist. High selectivities of an aza-Baylis-Hillman reaction with a CIL as reaction medium were reported by Leitner and coworkers, while Wasserscheid and coworkers demonstrated chirality transfer in a CIL with prochiral cations and chiral anions in asymmetric hydrogenation reactions [2,11,13]. A better understanding of ion aggregation and chirality transfer in CILs is necessary to enable the rational design of new catalytically active ionic liquids (ILs). Computer simulations can be a valuable tool in this respect since they offer a molecular view on ion aggregation in solution.

Many examples of successful chirality transfer experiments employ axially chiral compounds [20,21], which renders them promising for the design of new CILs. The rotational barrier of axially chiral *tropos* compounds is below 22.3 kcal/mol, meaning they can racemize at room temperature. The term racemization describes the irreversible conversion of an optically active compound (due to surplus of one enantiomer) into an optically inactive compound, where equal amounts of enantiomers are present [24]. If the rotational barrier of axially chiral compounds is greater than 22.3 kcal/mol, enantiomers are stable for more than 15 min at room temperature [25,26] and are called atropisomers [27], stemming from the ancient Greek word for “atropos” = “immutable, inflexible” [28]. Ligand and receptor chirality have a significant impact on biomolecular interactions, which is particularly essential for drug design, since two different enantiomers of a drug can differ significantly in their biological activity [29]. LaPlante et al. [25] discriminated between three classes of axial chirality based on the height of the rotational barrier: Class I molecules possess a barrier of less than 20 kcal/mol, which corresponds to a racemic mixture at room temperature in experiment. Class II molecules have a torsional barrier between 20 kcal/mol and 30 kcal/mol, which makes them problematic for drug design as the racemization can take place from within several minutes up to a month. This also hampers experimental analysis since the conversion between enantiomers can occur during measurements. However, class I and II molecules could be potentially interesting for chirality transfer as the switch from $R_{a}$ to $S_{a}$ or vice versa induced by the counter-ion may happen on a time scale of reactions. Class III molecules with rotational barriers higher than 30 kcal/mol are optimal for drug design as the racemization would take several years. In other words, the $R_{a}$ and the $S_{a}$ enantiomer can be synthesized separately. Class I molecules are *tropos*, while Class II and Class III molecules are atropisomers. Apart from being important for drug development, the use of atropisomeric, simple 1,1’-Bi-2-naphthol (BINOL)-derived chiral compounds already found a tremendously broad range of application in the field of asymmetric synthesis. In 1979 Noyori and co-workers published the use of a BINOL-based chiral reagent (BINAL-H) for asymmetric hydrogenations [30]. Since then, the classical BINOL-derived Class III compounds were proven to be extremely efficient for a large number of reactions as asymmetric catalysis as well [31]. Recently, another hot topic emerged in the field of asymmetric catalysis using atropisomeric and enantiomerically pure phosphoric acids. Starting from the first years of the 21st century, this field is constantly growing, providing a good alternative for a wide range of asymmetric transformations [32,33,34].

**Figure 1:**
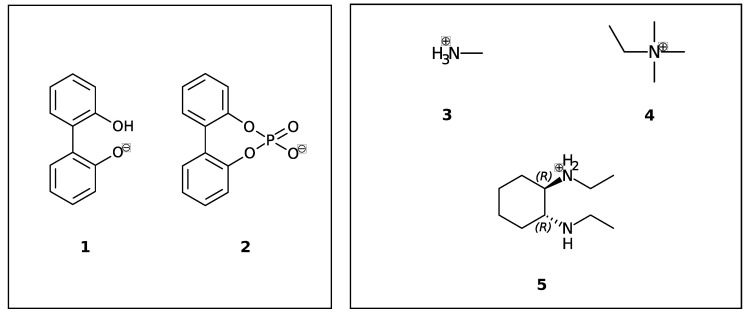
Axially chiral biphenyl-based anions (**1**, **2**) and achiral (**3**, **4**) and chiral cations (**5**) investigated in this study.

In this study, we are interested in the interconversion between enantiomers (enantiomerization [24]) of anionic biphenyls **1** and **2** (see Figure 1) in gasphase, *n*-butylmethylether (BuOMe) and water.

**Figure 2:**
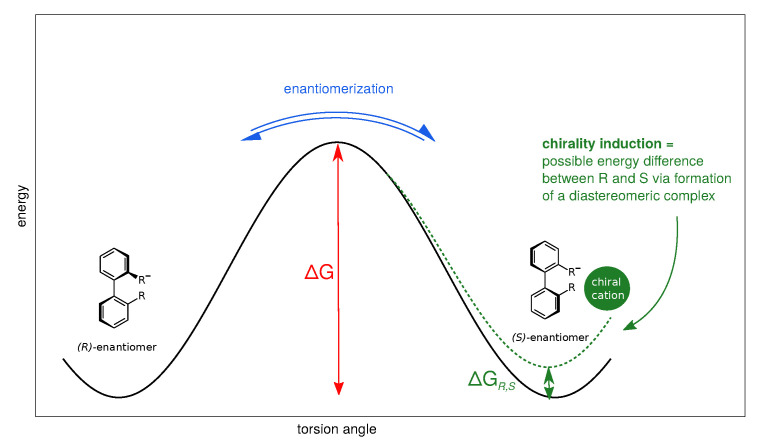
Schematic free energy profile of the enantiomerization in axially chiral biphenyl anions. The interconversion barrier ΔG determines the enantiomerization rate. If one conformer becomes energetically more favourable, e.g., due to formation of a diastereomeric complex with a chiral counterpart, the profile is rendered asymmetric (dashed line), resulting in two different diastereomerization barriers.

Anionic biphenyl **1** was chosen because experimental studies suggest chirality induction between its neutral form and a chiral diamine [35,36]. The phosphate bridged anion **2** was successfully used for selectivity enhancement in asymmetric transfer hydrogenation of enones [37]. In addition to the influence of the solvent, we want to investigate the effect of achiral and chiral cations on the enantiomerization barrier of the biphenyl anions **1** and **2**. We want to test if diastereomeric complex formation with a chiral cation can induce preference of one anion conformer, which would result in two diastereomerization barriers instead of a single enantiomerization barrier [38]. Figure 2 schematically illustrates the free energy profile of the enantiomerization process, and how chiral induction would be reflected as an asymmetry in the profile.

Despite their differing rotational barriers (see Results Section), both chiral anions **1** and **2** belong to Class I and are thus ideal candidates for the analysis of chirality transfer as the barrier may be overcome at room temperature. The amino-functionalized cations **3** and **4** are achiral and differ in their capability of hydrogen bonding to the anion. As already mentioned before, the neutral analog of cation **5** has been successfully applied in chirality transfer experiments [36].

Although the analysis of the rotational barriers of the biphenyls can be performed on a quantum-mechanical level, the enantiomerization process in various solvents is out of reach with this level of theory. Since we want to investigate the effect of hydrogen bonding between the distinct hydrogen bonding sites of the anion to both cation and solvent, explicit treatment of the solvent is preferable over dielectric continuum models. Hence, we augment our quantum-mechanical analysis of the rotational barrier with polarizable molecular dynamics simulations using umbrella sampling [39] to study the influence of the cations and the solvent on the interconversion.

## 2. Results

This study is organized in two parts: First, we present the generation of quantum-mechanical (QM) target data and construction of force fields from the target data for ortho-substituted biphenyl anions. Second, we apply these force fields in molecular dynamics (MD) simulation to study ion pairing and chirality induction of single ion pairs in solution.

### 2.1. Enantiomerization Barriers of Ortho-Substituted Biphenyls

Since for axially chiral biphenyl derivatives the transition between enantiomers proceeds via rotation around the aryl-aryl bond, it is crucial that the corresponding torsional energy profile is represented correctly in the force field, which is not the case for many standard force fields of unsubstituted biphenyl [40,41]. Our force fields are based on the CHARMM Drude force field [42], in which the general approach for fitting flexible degrees of freedom is to target QM scans on the MP2/6-31G(d) (6-31+G(d,p) for ions) level of theory. In the case of biphenyl-based anions **1** and **2**, care must be taken when generating QM target data in that way for several reasons: First and foremost, the QM treatment of biphenyl-based structures is not straightforward. Computing rotation barriers for simple unsubstituted biphenyl has been a particular challenge addressed in several studies [43,44,45,46,47], and has only been resolved in 2008 by Johansson and Olsen [41,48] by using a high-level coupled-cluster approach combined with several extrapolation schemes. The situation seems less challenging for substituted biphenyls, since good results have been obtained with standard DFT and ab initio approaches [49,50,51] in some cases. However, most of these studies investigate only small sets of specific biphenyls, and do not allow to make conclusions about the general applicability of specific QM methods for substituted biphenyls. To the best of our knowledge, the most systematic computational study on rotational barriers in substituted biphenyls so far was published by Masson in 2013 [52]. In this study, barrier energies of 13 substituted biphenyls computed with different density functional theory (DFT) methods are compared against the experiment. The author found that the B3LYP and B97 functionals augmented by an empirical dispersion correction [53,54] and a large triple-zeta basis set (def2-TZVPP [55]) gave the best agreement with experiment.

**Figure 3:**
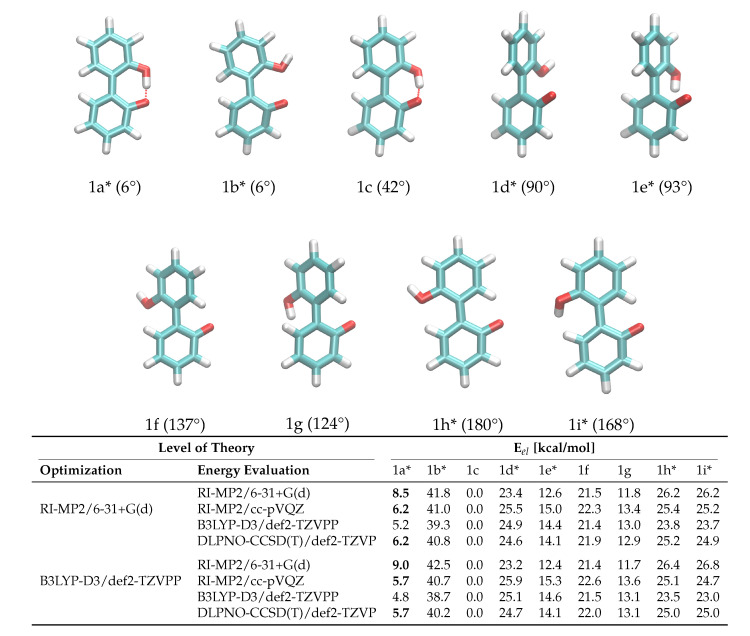
Ground and transition state geometries and their relative energies of anion **1** for the rotation around the aryl-aryl bond. The value in brackets is the corresponding dihedral angle. Transition states are marked with an asterisk (*). Only one enantiomer is shown for every state.

Another crucial prerequisite for precise barrier energies is the accuracy of transition state geometries. In ortho-substituted biphenyls, steric repulsion between the substituents can influence the transition state. When ortho-substituents have rotational degrees of freedom, different transition states depending on the rotational conformation of the ortho-substituent are possible (see Figure 3), which is the case for anion **1**. The rotational position of the hydroxy-hydrogen influences the energy of the transition states and, consequently, a simple one-dimensional dihedral scan does not suffice to describe the enantiomerization pathway and a two-dimensional scan exploring both the aryl-aryl dihedral as well as the rotation of the hydrogen, respectively, is necessary. However, in case of **1**, this 2D potential energy surface exhibits significant energy jumps because additional degrees of freedom change abruptly for particular conformations. For example, the strong intramolecular hydrogen bond causes significant changes in the O-H bond length and distortions in the geometry when the bond is broken during rotation. For **2**, the rotation cannot be described with one degree of freedom either, since the O-P-O-C and C-C-O-P dihedrals all change during the rotation. Consequently, we constructed the potential energy surface from QM ground and transition state optimizations without constraints (transition state 1b was omitted from the fit for **1** due to the high barrier). This gives fewer but more reliable points on the potential energy surface associated with the enantiomeric transition. The geometries of all ground and transition states optimized on the RI-MP2/6-31+G(d) level of theory are depicted in Figure 3. Although interconversion will likely proceed via the lowest-lying path and will not include all structures depicted in Figure 3, optimizing all possible ground and transition states is necessary in order to identify the most favorable pathway.

To probe the performance of different functionals and basis sets, we have optimized the structures in gas phase using two different levels of theory (RI-MP2/6-31+G(d) and B3LYP-D3/def2-TZVPP) and subsequently performed single point calculations on both structure sets using four different methods (listed in Figure 3). RI-MP2/6-31+G(d) and RI-MP2/cc-pVQZ were chosen as these methods are parts of the CHARMM Drude force field parametrization philosophy, and B3LYP-D3/def2-TZVPP was successfully applied by Masson in his work [52]. The DLPNO-CSSD(T)/def2-TZVP [56] level of theory is employed as a reference since we have used this level of theory successfully in a previous study [37]. The results are listed in Figure 3. It is of particular importance that the energy difference between ground state 1c and transition state 1a* is correctly reproduced since it is the lowest barrier through which interconversion can occur. Based on the data in Figure 3, it is clear that the RI-MP2/6-31+G(d) level is not sufficient to represent the barrier properly since it is about 40% higher compared to the DLPNO-CCSD(T)/def2-TZVP reference energy in the case of the RI-MP2 optimized geometries, and even 60% higher in case of the B3LYP-optimized geometries. The B3LYP-D3/def2-TZVPP barrier is lower by 1 kcal/mol, while barriers on the RI-MP2/cc-pVQZ and DLPNO-CCSD(T)/def2-TZVP level of theory coincide, justifying the use of RI-MP2/cc-pVQZ energies as target values for the force field fitting. Furthermore, the obtained geometries show a dependence on the optimization method with energies differing by approximately 0.5 kcal/mol. Since we aim at consistency with the existing CHARMM Drude force field, we will use the geometries optimized on the RI-MP2/6-31+G(d) level of theory. It is visible from the data that the lowest-energy pathway for the full rotation proceeds with the hydroxy-hydrogen pointed towards the second ring.

**Figure 4:**
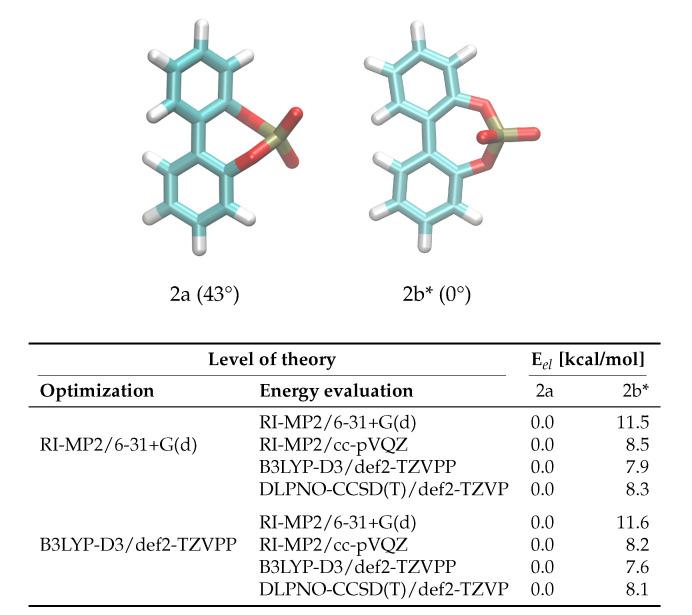
Ground and transition state geometries and their relative energies of anion **2** for the rotation around the aryl-aryl bond. The value in brackets is the corresponding dihedral angle. The transition state is marked with an asterisk (*). Only one enantiomer is shown for every state.

The enantiomerization pathway for anion **2** is comparably simple, with the bridged phosphate group allowing only one possible transition state (Figure 4). The same QM analysis as for anion **1** has been performed for anion **2**, with the resulting energies being shown in Figure 4. In contrast to compound **1**, the level of theory used for the optimization is not as significant as for **1**, with the most substantial difference being 0.3 kcal/mol between barriers. However, it is also clear that RI-MP2/6-31+G(d) gives insufficient energies that are about 40% higher than the DLPNO-CCSD(T)/def2-TZVP results. The B3LYP-D3/def2-TZVPP energies are closer to the DLPNO-CCSD(T)/def2-TZVP barriers than for **1**, and again the RI-MP2/cc-pVQZ level of theory gives satisfying results that can be used in the force field fit.

Both anions definitely belong to class I of axially chiral molecules concerning the rotational barrier at 0${}^{\circ }$. The lowest barriers are less than 10 kcal/mol and thus more or less frequently crossed during polarizable MD simulations.

### 2.2. Construction of the Polarizable Force Field

Using the generated QM data on the RI-MP2/cc-pVQZ//RI-MP2/6-31+G(d) level of theory as target data, dihedral potentials associated with the rotation around the aryl-aryl-bond were fitted. To generate molecular mechanics (MM) energies, the aryl-aryl dihedral in both anions, as well as the dihedral associated with rotation of the ortho-substituent in **1** were held fixed at values obtained from the QM geometry optimizations and all other remaining degrees of freedom were minimized. Improper dihedrals were added to prevent unwanted out-of-plane-bending, which allowed exploring the enantiomerization pathway along this one degree of freedom. For **1**, the aryl-aryl dihedral parameters as well as the H-O-C-C dihedral parameters were fitted. For **2**, the aryl-aryl dihedral parameters and the C-O-P-O and C-C-O-P dihedral parameters were fitted to reproduce the torsional profile. The parameter values can be found in Supplementary Material 8.

**Figure 5:**
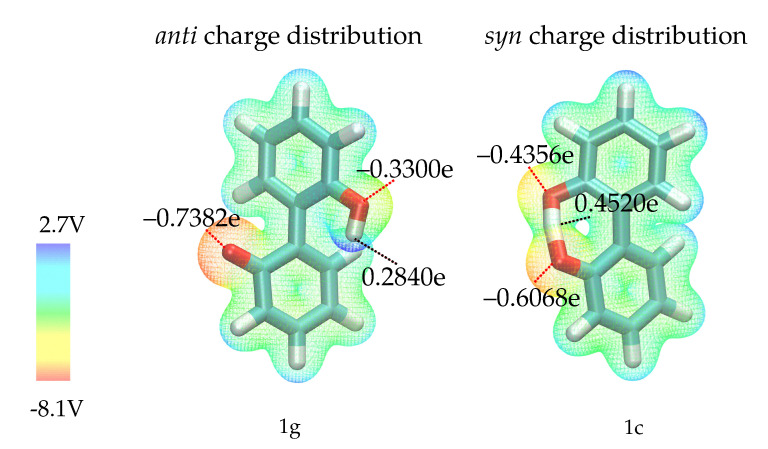
Molecular electrostatic potential for geometries 1g (**left**) and 1c (**right**), computed on the RI-MP2/Sadlej-TZ level of theory and mapped to the electron density surface at 0.002 e/Å${}^{3}$. Please note that the shown partial charges are the charges used in the force field, which differ from the QM partial charges since polarizable force fields with Drude particles require refitting of the QM charges.

Before discussing the fit results, it is necessary to analyze the effect of the intramolecular hydrogen bond in anion **1**. Via this hydrogen bond, charge can be transferred from the negatively charged oxygen, resulting in different charge distributions in the most favorable *syn* and *anti* ground states (1c and 1g in Figure 3) as depicted in Figure 5. Since the *syn* ground state (containing the hydrogen bond) is preferred by about 13 kcal/mol, it is unlikely that the *anti* conformation is visited in simple equilibrium simulations. However, since we want to explore the full rotation around the aryl-aryl bond and cannot exclude competitive hydrogen bonding of other ions, we need to consider that the charge distribution will change once the hydrogen bond is broken. To investigate the effect of the two different charge distributions, we have constructed two different force fields for compound **1** -one uses partial charges computed on the geometry of 1c, the other one charges computed on the geometry of 1g. The two different partial charge distributions will be labelled as *anti* (distribution corresponding to geometry 1g) and *syn* (distribution of geometry 1c) throughout the text. As the hydroxy-proton is partially shared by both oxygens in the *syn* configuration, the negative partial charges of the oxygens are more similar compared to the *anti* configuration. Here, the oxygen without the attached hydrogen is far more negatively charged. Consequently, a stronger intramolecular hydrogen bond is expected for the *anti* partial charge distribution (see left Figure 5).

**Figure 6:**
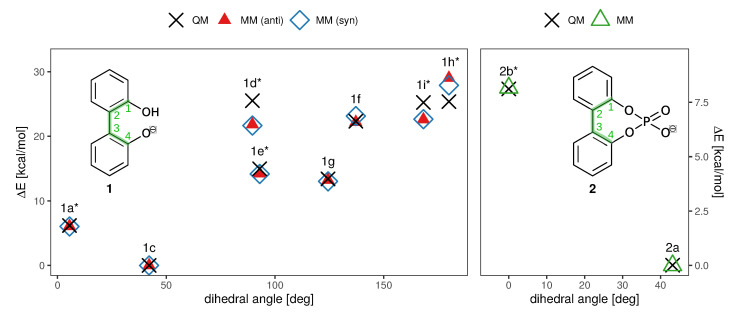
QM and MM torsional profile of compounds **1** and **2**, constructed from the structures shown in Figure 3 and Figure 4. For **1**, results for both the *syn* and *anti* charge set are depicted. The dihedral plotted on the x axis is marked in green in the insets. Points represent the conformations shown in Figure 3 and Figure 4 and are labelled accordingly. Again, transition states are marked with an asterisk (*). Since the profiles are symmetric, only one half is shown.

The results of the torsional fit for both charge distributions are depicted in Figure 6. While the lower energy differences are well captured by the fitted parameters, higher-energy parameters are not reproduced as well but play a minor role in enantiomerization due to their much higher energy compared to the 0${}^{\circ }$ barrier. As rotation is most likely to proceed via the lowest-energy pathway, the resulting fit is satisfying. Consequently, in the simulations the torsional potential will only be computed from −160${}^{\circ }$ to 160${}^{\circ }$ for anion **1**. In case of the bridged anion, the scan is restricted from −60${}^{\circ }$ to 60${}^{\circ }$.

### 2.3. Effect of the Solvent

**Figure 7:**
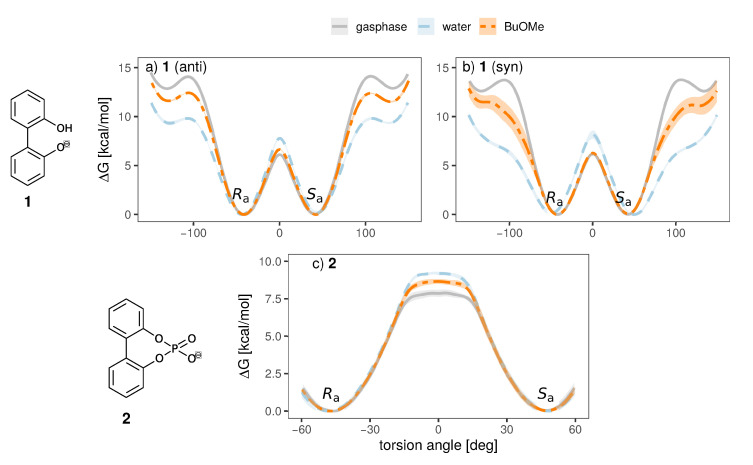
Free energy torsional profile of the aryl-aryl bond rotation in **1** (**a**,**b**, **top panel**) and **2** (**c**, **bottom panel**), for a single molecule in gasphase (grey) and a single ion pair with cation **3** in water (blue) and BuOMe (orange). For **1**, profiles obtained with both charge distributions (*anti* in **a**, *syn* in **b**) are shown.

To investigate the influence of the solvent and counterion on the enantiomeric stability of the anion, we have conducted umbrella sampling simulations to compute the free energy profile of rotation around the aryl-aryl bond (10${}^{\circ }$ increments, additional windows at −15, −5, 5 and 15${}^{\circ }$ for anion **2**, see Figures S1 and S2 for a more detailed discussion). To investigate the effect of the solvent, we will investigate systems of a single ion pair of cation **3** and one anion (either **1** or **2**) in water and *n*-butylmethylether (BuOMe). In Figure 7, the free energy torsional profile of the rotation around the aryl-aryl-bond of the anion is shown for a single ion pair of cation **3** together with anion **1** and **2**, respectively (gasphase profile is single anion only). The shaded areas depict the standard deviation of the respective energy profile.

It is clearly visible that the solvent strongly influences the profile. The 0${}^{\circ }$ barrier, which is the lowest and hence the most important one, is increased in water compared to the less polar BuOMe and gasphase. Contrary, the barriers at 90${}^{\circ }$ are lowered by approximately 2.5 kcal/mol compared to BuOMe, which is likely caused by hydrogen bonding with water molecules. The influence of solvent on rotational barrier energies has been reported in literature [57,58]. Demir-Ordu et al. observed an increase in barrier energies of an axially chiral compound by hydrogen bonding to the solvent [59]. QM studies on ortho-substituted biphenyls by Masson [52] suggest that solvent effects are even more pronounced when the biphenyl compound bears charged substituents in the ortho-positions, such as the biphenyls used in this study. The effect of water on the torsional barrier is stronger for the *syn* partial charge distribution (see Figure 7a,b). Furthermore, the minima of the torsional potential are slightly shifted in water for the *syn* configuration by 15${}^{\circ }$ to higher absolute angles (see Figure 7b). Thus, the interaction of the *syn* partial charge distribution with water seems stronger.

Interestingly, the torsional barrier at 0${}^{\circ }$ of the phosphoric acid bridged anion **2** in water is only 1 kcal/mol higher than the corresponding barrier of the biphenolat **1**. Apparently, the ring tension is not significantly higher compared to the intramolecular hydrogen bond of compound **1**. Additionally, the rotational barrier of **2** exhibits a small plateau which is broader compared to **1**.

**Figure 8:**
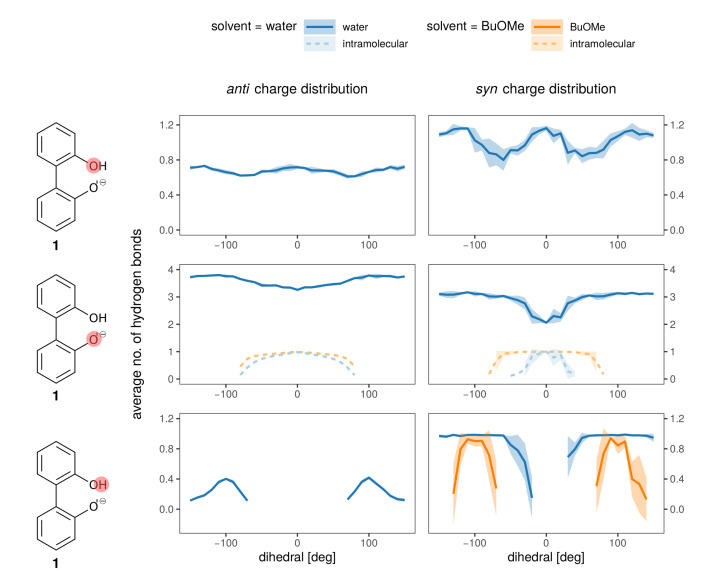
Hydrogen bonding to all hydrogen bonding sites of the anion (single ion pair of **1** and **3** in solvent).

Hydrogen bonds are most probably the strongest interaction between the anions **1** and **2** and the solvent. In Figure 8 the average number of hydrogen bonds is depicted for the oxygens and the hydroxy-proton of **1**. Hydrogen bonds to the solvent (blue for water, orange for BuOMe) are displayed as solid lines, whereas the intramolecular hydrogen bond is shown as a dashed line. As expected from the energy profiles in Figure 7a,b, the number of hydrogen bonds between the *syn* compound and water shows a stronger dependence on the dihedral angle and consequently, the rotational barrier changes. However, the average number of hydrogen bonds to the oxygens is roughly the same for *anti* and *syn*. The intramolecular hydrogen bond between −60${}^{\circ }$ and 60${}^{\circ }$ seems to be neither a function of the dihedral angle nor of the solvent as visualized by the dashed lines in the middle of Figure 8. Butylmethylether can only accept hydrogen bonds. The only hydrogen which may act as hydrogen donor of **1** is the hydroxy proton. The hydrogen is a much stronger donor in the *syn* charge distribution, as it carries a higher partial charge (0.4520e compared to 0.2840e). This difference in hydrogen bonding likely causes the discrepancy between the *syn* and *anti* profiles for water in Figure 7, also because the position of the hydrogen influences the rotational barrier as shown in Figure 3.

In anion **2**, hydrogen bonds to the solvent are only possible with water, and there is no dependence on the dihedral angle. The oxygens adjacent to the rings form on average 0.6 hydrogen bonds with water, while the terminal oxygens form 2.4 on average (data shown in Figure S6).

### 2.4. Effect of the Counterion

The initial idea behind using cation **5** was to explore a possible chirality transfer between cation and anion, since experimental studies on successful chirality transfer between the neutral analogs of chiral cation **5** and anion **1** exist [36]. In these studies, the authors suggest formation of a complex where the neutral form of **5** acts as a “bidentate“ ligand that accepts hydrogen bonds from the hydroxy groups of the neutral form of **1**. Although anion **1** can donate only one hydrogen bond and intramolecular bonding is stronger in the ionic form, we nevertheless want to explore the possibility of chiral induction, since charged compounds drastically increase the chance of complex formation. Furthermore, the cation acts as a stronger hydrogen bond donor due to its free protic hydrogens. If a diastereomeric complex between the chiral cation **5** and anion in which either $R_{a}$ or $S_{a}$ is preferred is formed, this should be visible in the free energy profile.

**Figure 9:**
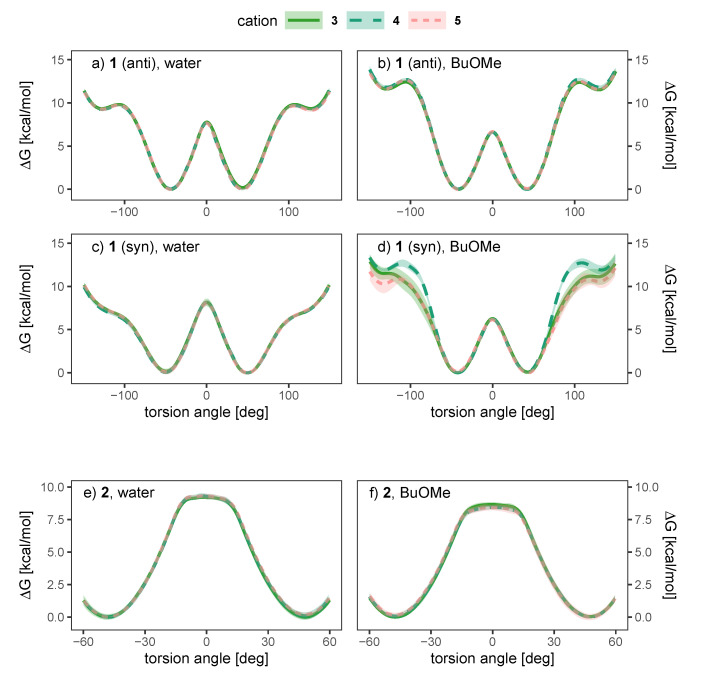
Dependence of the free energy torsional profile of anions **1** (*anti* (**a**,**b**, **top**) and *syn* (**c**,**d**, **middle**) charge distribution) and **2** (**e**,**f**, **bottom**) on the counterion, for a single ion pair in water (**a**,**c**,**e**, **left**) and BuOMe (**b**,**d**,**f**, **right**).

As water is a polar solvent with a dielectric constant of roughly 80, the probability of ion pairing is low as each ion is surrounded by its own hydration shell. Consequently, we expect only weak chirality transfer. As shown in Figure 9a,c the dihedral potential of the anion **1** is symmetric in water and does not depend on the cation, since simulations containing the achiral cations **3** and **4** result in the same free energy profile as the simulation using the chiral cation **5**. The picture does not change in the apolar BuOMe, which has a dielectric constant of 4.5 and consequently a much higher probability for ion pairing. For the *anti* charge distribution, the free energy profile is still not a function of the cation as shown in Figure 9b,d. For the *syn* charge distribution, there is a slight cation dependence of the profile in the regions around −100${}^{\circ }$ and 100${}^{\circ }$, but the profile in between is similar for all three cations. The profiles are still symmetric, indicating no preference of either enantiomer. These results suggest that hydrogen bonding of the anion to the cation plays a negligible role, as the achiral cation **4** cannot form hydrogen bonds in contrast to **3**. For anion **2**, no notable influence of the cation on the profiles can be detected in Figure 9e,f either, and as for compound **1**, the profiles indicate no preference for one enantiomer.

**Figure 10:**
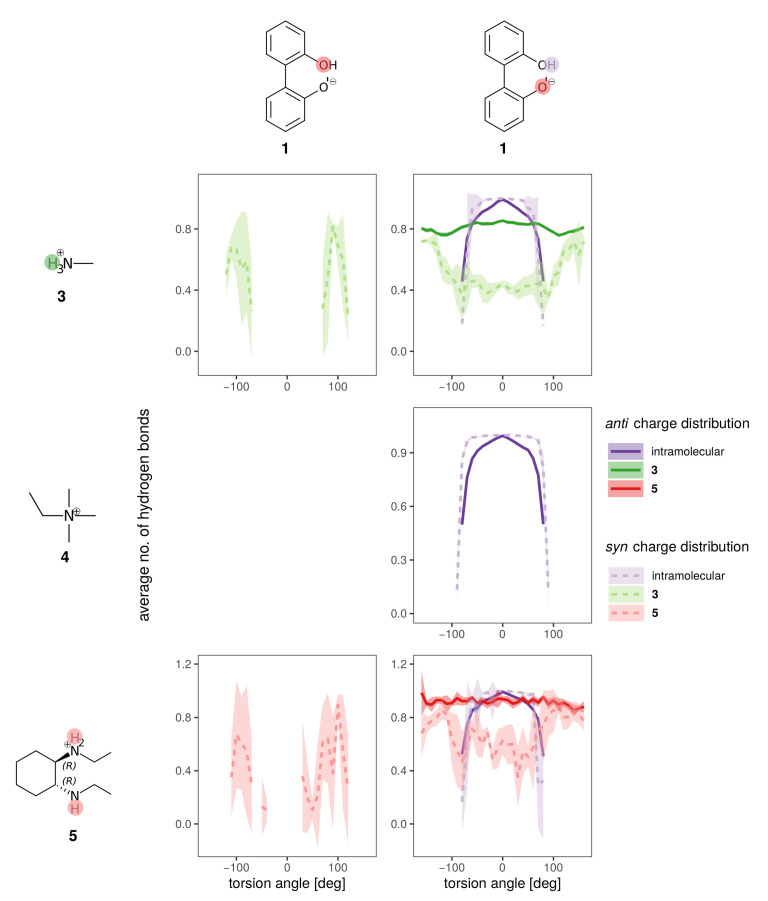
Hydrogen bonding of anion **1** for the *syn* (lighter dashed lines) and *anti* charge distribution (darker solid lines) for the different counterions in BuOMe.

The hydrogen bonding of both charge distributions of anion **1** to the cations is shown in Figure 10 for the BuOMe solution. As already discussed, **4** cannot form hydrogen bonds and consequently only the intramolecular hydrogen bond of biphenyl **1** is depicted. The intramolecular hydrogen bond (violet dashed and solid lines) is similar for both charge distributions, and is hardly affected by cation choice. The intermolecular hydrogen bond between the anion and cations **3** and **5** is still not a function of the cation, but there are some differences between the *syn* (dashed lines) and *anti* charge distribution (solid lines).

As the partial charges of the oxygens for the *syn* charge distribution differ less compared to the *anti* (see Figure 5), the overall U-shaped hydrogen bonding profile of the *syn* oxygens as a function of the dihedral angle resembles each other. Only the minimum for the hydroxy-oxygen is lower as no hydrogen bonding occurs around 0${}^{\circ }$. In case of the anionic oxygen, the minimal number of hydrogen bonds is still 0.4 at 0${}^{\circ }$. The situation drastically changes for the *anti* charge distribution. Here, the hydroxy-oxygen has zero hydrogen bonds to the cations irrespective of the dihedral angle. The anionic oxygen with the much more negative partial charge (−0.7382e) forms 0.8 hydrogen bonds on average to the cations which is also not a function of the dihedral angle. The strongest change in the hydrogen bonding as a function of the dihedral angle can be found at −100 and + 100${}^{\circ }$ for the *syn* charge distribution. At the same angles, the deviations in the free energy profiles in Figure 9d occur indicating that the hydroxy oxygen plays a major role.

Only the terminal oxygens of the anion **2** form hydrogen bonds to the cations **3** and **5**. The number of hydrogen bonds is not a function of the dihedral angle and on average, 0.45 and 0.5 hydrogen bonds exist, respectively (data shown in Figure S7).

## 3. Concluding Discussion

For the analysis of the enantiomerization of axially chiral biphenyls in solution, a dual method approach is preferable. If multiple degrees of freedom are necessary to describe the aryl-aryl torsion in a QM scan, ground and transition state opimizations without restraints can be used to construct the potential energy surface. To obtain accurate barrier energies for substituted biphenyls, high-level methods such as the RI-MP2/cc-pVQZ level used in this study are necessary. Since the influence of the solvent and cation are out of reach for high-level quantum-mechanical methods, we used the QM data to parametrize a polarizable force field. In molecular dynamics simulations the free energy ΔG of the aryl-aryl torsion as a function of the cations and solvent is accessible. In this work, we have shown that the solvents *n*-butylmethylether and water affect the free energy profile of the torsion. In water, the lowest energy barrier at a dihedral of 0${}^{\circ }$ is increased compared to *n*-butylmethylether. However, the nature of the cations has only a marginal impact on ΔG(ϕ), and symmetric profiles are obtained in all cases. We therefore conclude that for the systems investigated in this work, no chirality transfer has taken place although the cations form hydrogen bonds to the anion. We have observed no preference for $R_{a}$ or $S_{a}$ in the simulations.

Another important factor in the force field development for biphenyl-based compounds is the influence of the configuration on the partial charge distribution, which is especially important for anion **1** due to the intramolecular hydrogen bond. In this study we have shown that the *syn* and *anti* configuration of **1** result in different partial charge distributions which influence the hydrogen bonding behavior. Although dihedral parameters were adapted independently to reproduce the target QM energies as closely as possible, different hydrogen bonding with the solvent results in different profiles, which is especially problematic when the simulation aims at exploring conformations that differ from the one used for generation of partial charges. As classical molecular dynamics simulations operate with fixed partial charges, a switch between *syn* and *anti* partial charge distribution would be desirable. However, since this is not viable in long-term molecular dynamics simulations, at least polarizable simulations are preferable as the induced dipoles may model the change of the electrostatic potential as a function of the aryl-aryl dihedral angle to some extent. Although the anions investigated in this work belong to class I of axially chiral molecules with rotational barriers of less than 20 kcal/mol, the computation of the free energy ΔG requires exceptionally long molecular dynamics simulations, as even simulations of more than 100 ns did not yield adequate statistics. Consequently, enhanced sampling techniques such as the umbrella sampling used in this study are strongly recommended.

## 4. Materials and Methods

### 4.1. Quantum-Mechanical Calculations

All QM calculations were performed with the quantum-chemistry package ORCA [60]. Ground and transition state geometry optimizations were performed on the the RI-MP2/6-31+G(d) (auxiliary basis for RI-MP2: def2-TZVP) and B3LYP-D3/def2-TZVP level of theory. In all calculations employing the B3LYP functional, correlation of the uniform electron gas was modeled according to the Vosko-Wilk-Nusair VWN5 formalism [61]. Transition state geometries were optimized in a two-step procedure: In the first step, a constraint minimization with a restraint on the aryl-aryl-dihedral (0${}^{\circ }$, 90${}^{\circ }$ or 180${}^{\circ }$ for the respective transition states) was performed, and the resulting structure used as input for unrestrained transition state optimization. Transition states were verified by subsequent frequency calculations. On the obtained geometries, single point calculations with a set of different methods and basis sets were performed with ORCA (see Figure 3 for an overview of the employed methods). For the RI-MP2/cc-pVQZ calculations, a cc-pVQZ auxiliary basis was used. The molecular electrostatic potentials shown in Figure 5 were computed with ORCA on the RI-MP2/Sadlej-TZ level of theory and mapped to the electron density surface at 0.002 e/Å${}^{3}$.

### 4.2. Force Field Parametrization

In all simulations, electronic polarizability was included via Drude oscillators. Parameters were based on the existing CHARMM Drude force field (see reference [42] and references therein) which is available from http://mackerell.umaryland.edu/charmm_drude_ff.shtml. Ions **3** and **4** as well as the solvents (*n*-butylmethylether BuOMe and the SWM4 water model) were readily available from this force field, ions **1**, **2** and **5** required additional parametrization.

The optimization targeted quantum-mechanically calculated reference data. All QM calculations were performed with ORCA [60] and PSI4 [62]. Parametrization proceeded in two main steps: In the first, electrostatic parameters (partial charges, atomic polarizabilities, Thole screening factors) were optimized, followed by optimization of bonded parameters. Lennard-Jones parameters were not optimized, but taken from the CHARMM Drude force field. Atomic polarizabilities and charges in gas phase were computed according to protocols described in detail in references [63,64,65] using geometries optimized at the RI-MP2/6-31+G(d) level of theory: Atomic polarizabilities were obtained on the RI-MP2/Sadlej-TZ level of theory using the methodology described in references [63,64,65]. According to suggestions in reference [42], polarizabilities were subsequently scaled by empirical factors, 0.85 for the cation, 0.724 for anion **1** and 0.6 for anion **2**. Initial Thole screening factors were taken from similar structures already included in the CHARMM Drude force field and subsequently modified to reproduce the components of the molecular polarizability.

Initial atomic charges were obtained via restrained electrostatic potential (RESP) fitting [66,67] on the RI-MP2/Sadlej-TZ [68] level of theory. Charges were then modified to reproduce the components of the molecular dipole moment as well as interaction energies of hydrogen bonding sites with water, all computed with single point RI-MP2/cc-pVQZ energy evaluations on structures optimized at RI-MP2/6-31+G(d) [69]. Compound 1 can form a relatively stable intramolecular hydrogen bond, which influences the charge distribution—to investigate this, two charge distributions were computed for **1**—one with and one without the hydrogen bond (*syn* and *anti*, see Figure 5 and Figure S5). A more thorough discussion of this issue can be found in the Results section.

In a second step, bonded parameters were optimized. To ensure compatibility with the existing CHARMM Drude force field, only parameters not already included in the force field were optimized. To alleviate the cost of parametrization, we assigned parameters by similarity as often as possible and explicitly fitted only those that have no resembling parameters and/or are crucial to the overall conformation of the molecule. Fitted parameters are marked explicitly in the force field (see Supplementary Material 8). For the parameters that were explicitly fitted, QM relaxed scans of the relevant degree of freedom on the RI-MP2/6-31+G(d) level of theory were conducted. Parameters were then fitted to target these scans using the lsfitpar program [70] as well as manual fitting in some cases. The aryl-aryl bond torsion of compounds **1** and **2** was fitted as described in the results section—the potential energy surface was constructed from ground and transition state optimizations without geometry restraints. The specific dihedrals fitted to reproduce the aryl-aryl torsion are marked in the force fields (see Supplementary Material 8). Optimizations were performed on the RI-MP2/6-31+G(d) level of theory, followed by energy evaluations on the RI-MP2/cc-pVQZ level of theory. Furthemore, improper dihedrals were added to prevent unwanted out-of-plane bending for the biphenyl-based compounds.

## 5. Umbrella Sampling

All simulations were performed with a GPU-capable version of the program package CHARMM [71,72]. In all simulations, the Velocity-Verlet integrator [73] with a timestep of 0.5 fs was employed. All simulations made use of a dual thermostat, using the Andersen-Hoover equations at constant pressure and the Nosé-Hoover equations at constant volume. The SHAKE algorithm was applied to fix lengths of bonds to hydrogen atoms [74]. Drude masses were set to 0.4 amu.

### 5.1. Gasphase Simulations

**Figure 11:**
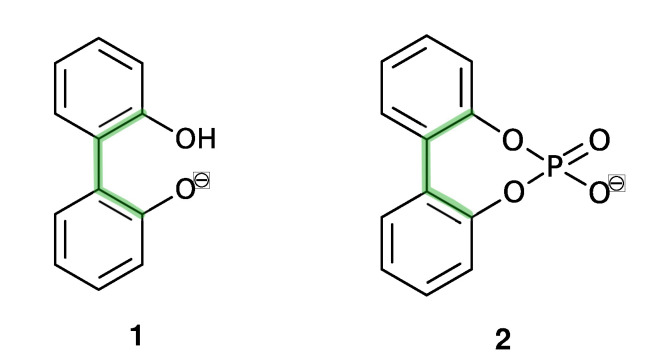
During production runs, the dihedral angles marked in green were held fixed at a certain value by a harmonic restraint.

For simulations of a single ion in gas phase at *T* = 300K, an infinite cut-off was applied and electrostatic interactions were calculated explicitly. For the Umbrella sampling [39], a harmonic restraint with a force constant of 100 kcal mol${}^{-1}$ rad${}^{-2}$ (CONS DIHE command in CHARMM) was placed on the torsion angles depicted in Figure 11.

Separate simulations at 10${}^{\circ }$ intervals were used to generate umbrella sampling data, with a range of −160${}^{\circ }$ to 160${}^{\circ }$ for anion **1** and a range of −60 to 60${}^{\circ }$ for anion **2**. For anion **2**, additional windows at −15, −5, 5, and 15${}^{\circ }$ were added (see Figure S1 for further discussion). After applying the restraint, each simulation was equilibrated for 250 ps, followed by a production phase of 5 ns (see Figure S3 for further discussion). Data was written to disk every 500 ps. The free energy profile was obtained in a post-processing step with the vFEP (variational free energy profile) program [75]. By splitting the data into five blocks and computing individual profiles for each block, the standard deviation was estimated.

### 5.2. Simulations in Solution

Simulations were performed at *T* = 300 K and atmospheric pressure. In all simulations, periodic boundary conditions were applied. A non-bonded cut-off of 12 Å and a smooth switching function between 10 and 12 Å were used. Electrostatic interactions were calculated with the Particle Mesh Ewald Method, employing a grid of approximately 1 Å, cubic splines of order 6 and κ=0.41 Å${}^{-1}$. For each system, five replica simulations were performed. Data for each replica was generated as follows: Initial configurations of a single ion pair in solution were generated with PACKMOL [76] and equilibrated in the NpT ensemble for 0.5 ns. Subsequently, the dihedral restraint was applied according to the protocol used in the gas phase. After applying the restraint, the system was allowed to equilibrate for 0.25 ns in the NVT ensemble, followed by a production phase of 1 ns in the NVT ensemble. Data was written to disk every 0.5 ps, resulting in 2000 data points per replica. 5 replica per window were generated this way, resulting in an overall simulation times of 5 ns (10,000 data points) per window, see Figure S4. An overview of the simulations can be fund in Supplementary Material 6. Dihedral values were extracted with CHARMM in a post-processing step. The free energy profiles were again computed with the vFEP program. Hydrogen bonds for every replica simulation were analyzed with CHARMM, using distance and angle cut-offs of 2.4 Å and 135 ${}^{\circ }$, respectively. Standard deviations for free energy profiles and hydrogen bonding were computed from the five replica simulations. Figures were created using VMD [77] and the ggplot2 package within the R program suite [78,79].

## Supplementary Materials

The following are available at https://www.mdpi.com/1422-0067/21/17/6222/s1.

## Abbreviations

The following abbreviations are used in this manuscript:

CIL	Chiral ionic liquid
QM	Quantum-mechanical
MM	Molecular mechanics
MD	Molecular dynamics
BuOMe	*n*-butylmethylether
