# Supplementary material for: Enantiomerization of Axially Chiral Biphenyls: Polarizable MD Simulations in Water and Butylmethylether
Source: Int J Mol Sci. 2020 Aug 28;21(17):6222. doi: 10.3390/ijms21176222 (PMC7503397; doi:10.3390/ijms21176222)
Supplement: Supplementary file 1 [file ijms-21-06222-s001.pdf]

Supplementary Material For “Enantiomerization of Axially Chiral Biphenyls: Polarizable MD Simulations in Water and Butylmethylether“

Contents

1 Choice of window size for gasphase simulations **2**

2 Choice of window size for solvent simulations **3**

3 Convergence of profiles in gas phase **4**

4 Convergence of profiles in solvent **5**

5 Generation of data for *syn* and *anti* charge distribution of anion 1 **6**

6 Overview of simulations in solvent **7**

7 Hydrogen bonding of anion 2 **8**

8 Force Fields **9**

8.1 Cation **5** 9

8.2 Anion **1**, *anti* charge distribution 13

8.3 Anion **1**, *syn* charge distribution 16

8.4 Anion **2** 19

1 Choice of window size for gasphase simulations

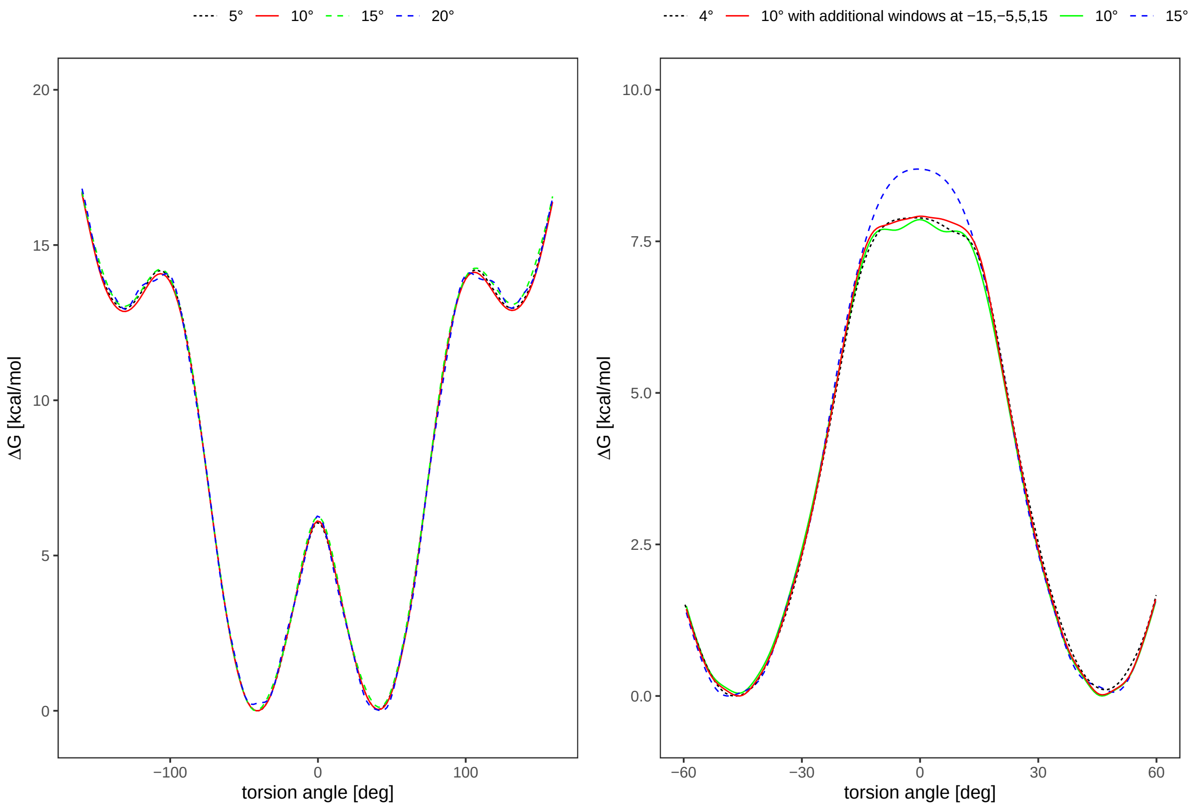

Figure **S1**: Gasphase free energy profiles (simulation time per window: 5 ns) of **1** (*anti* charge distribution, left) and **2** (right) at 300 K using different window sizes.

To determine suitable window sizes for the umbrella sampling simulations, gas phase umbrella sampling simulations of a single ion at different window sizes were conducted. Overlap of histograms was visually checked to determine a suitable range for initial window sizes. Profiles were then calculated with the vFEP program, which requires less overlap between windows than other free energy estimation methods [1]. The respective profiles at different window sizes are shown in Figure **S1** (anion **1** (*anti* charge distribution) left, anion **2** right). For **1**, only the *anti* charge distribution was simulated to reduce cost. It is visible that the profiles for **1** are overlapping, but the profile at $\Delta\phi = 20°$ is not smooth in the -100 and 100 degree regions. The profile at $\Delta\phi = 15°$ is smoother, but still not fully coinciding with the $\Delta\phi = 5°$ and $\Delta\phi = 10°$ profiles at $0°$. The $\Delta\phi = 5°$ and $\Delta\phi = 10°$ profiles are fully coinciding, hence a window spacing of $10°$ was chosen.

For anion **2**, all profiles seem to overlap in the minima regions, but agree poorly around the maximum. From the histograms, it was visible that overlap was poor in this region when larger windows were used, so a $4°$ was used as a reference. The $\Delta\phi = 15°$ profile is much too high around the maximum, and the $\Delta\phi = 10°$ profile shows irregularities in this region. By introducing additional windows at -15,-5,5 and 15 °, convergence with the $\Delta\phi = 4°$ profile could be achieved.

2 Choice of window size for solvent simulations

The dependence on window size in solvent was evaluated for single ion pairs of **3** combined with **1** (*anti* charge distribution) and **2**, respectively. The data is shown in Figure S2. Due to the increased cost of solvent simulations, only two window sizes were tested as an educated guess could already be made from the gasphase simulations. From Figure S2, it is visible that the final window sizes identified in the gas phase simulations (10° for **1** and 10° with additional windows at -15, 5, 5, 15° for **2**) are also suitable for solvent simulations.

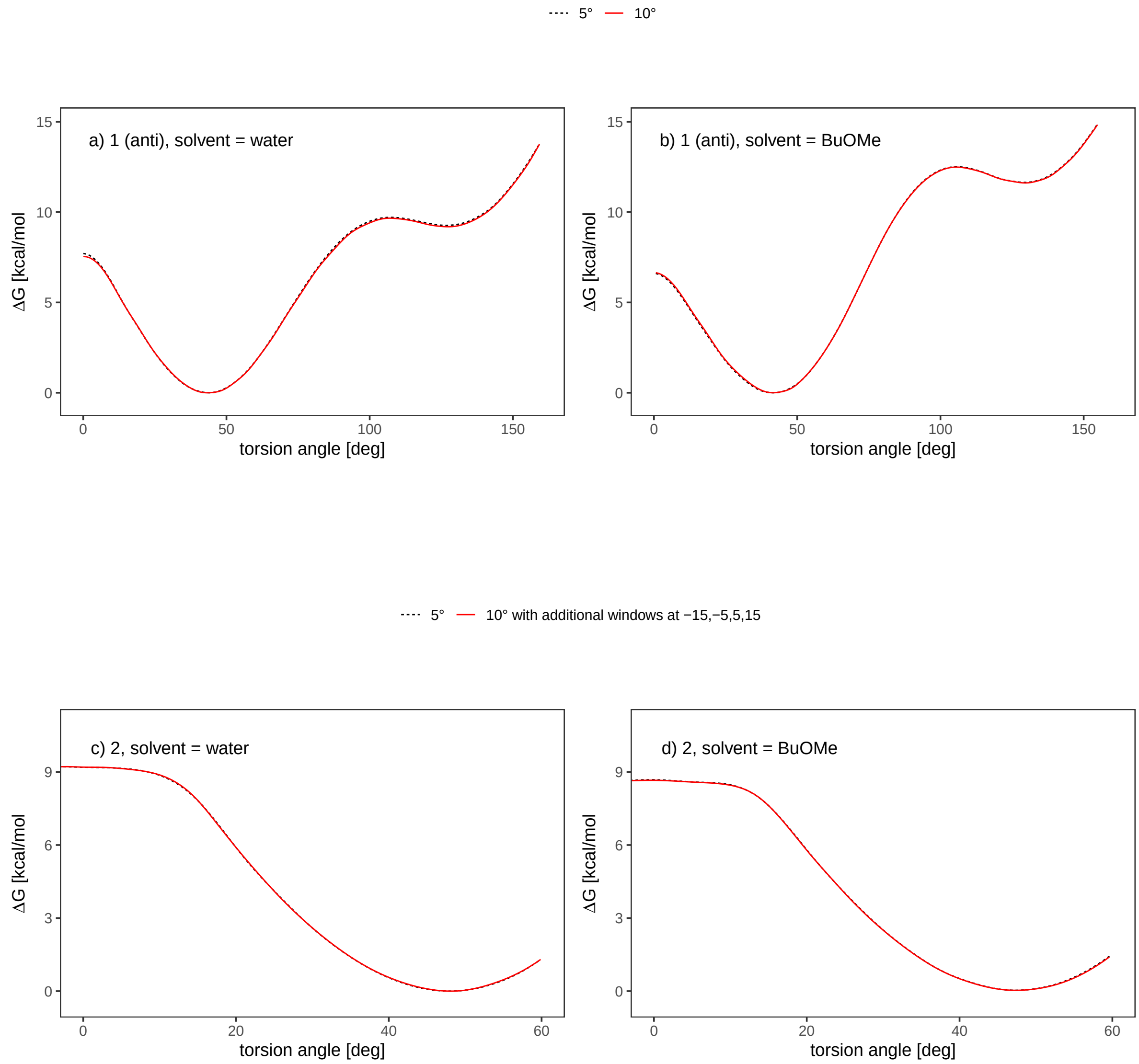

Figure **S2**: Free energy profiles in solvent (simulation time per window: 5 ns) of **1** (*anti* charge distribution, top) and **2** (bottom) at 300K using different window sizes. Since the profiles are symmetric, only the profiles from 0 to 180° and 0 to 60° were computed.

3 Convergence of profiles in gas phase

Figure S3 shows the dependence of the free energy profiles on the total simulation time per window for a single anion in gas phase. For both charge distributions of **1**, profiles are already converged after the shortest simulation time of 1 ns. For anion **2**, full convergence is only achieved after 3 ns. 5 ns of simulation time were chosen to achieve reasonable standard devations (depicted in main manuscript).

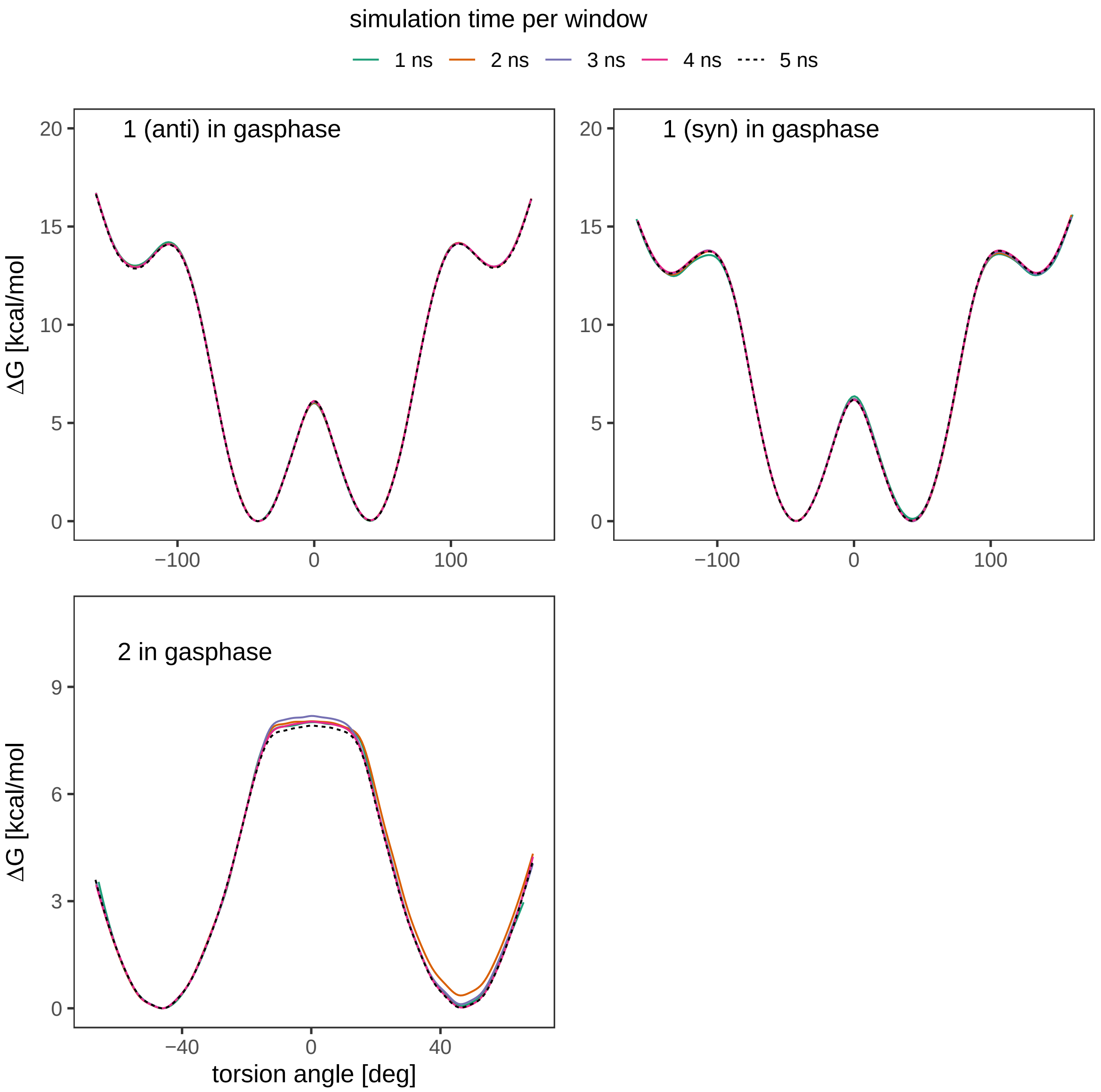

Figure **S3**: Free energy profiles of a single ion in gas phase computed with different simulation lengths.

4 Convergence of profiles in solvent

Figure S4 shows the dependence of the free energy profiles on the total simulation time per window for a single ion pair of cation **3** and anion in water and BuOMe. All profiles are converged after 5 ns/window, and 5 ns were chosen to achieve reasonable standard deviations (depicted in main manuscript).

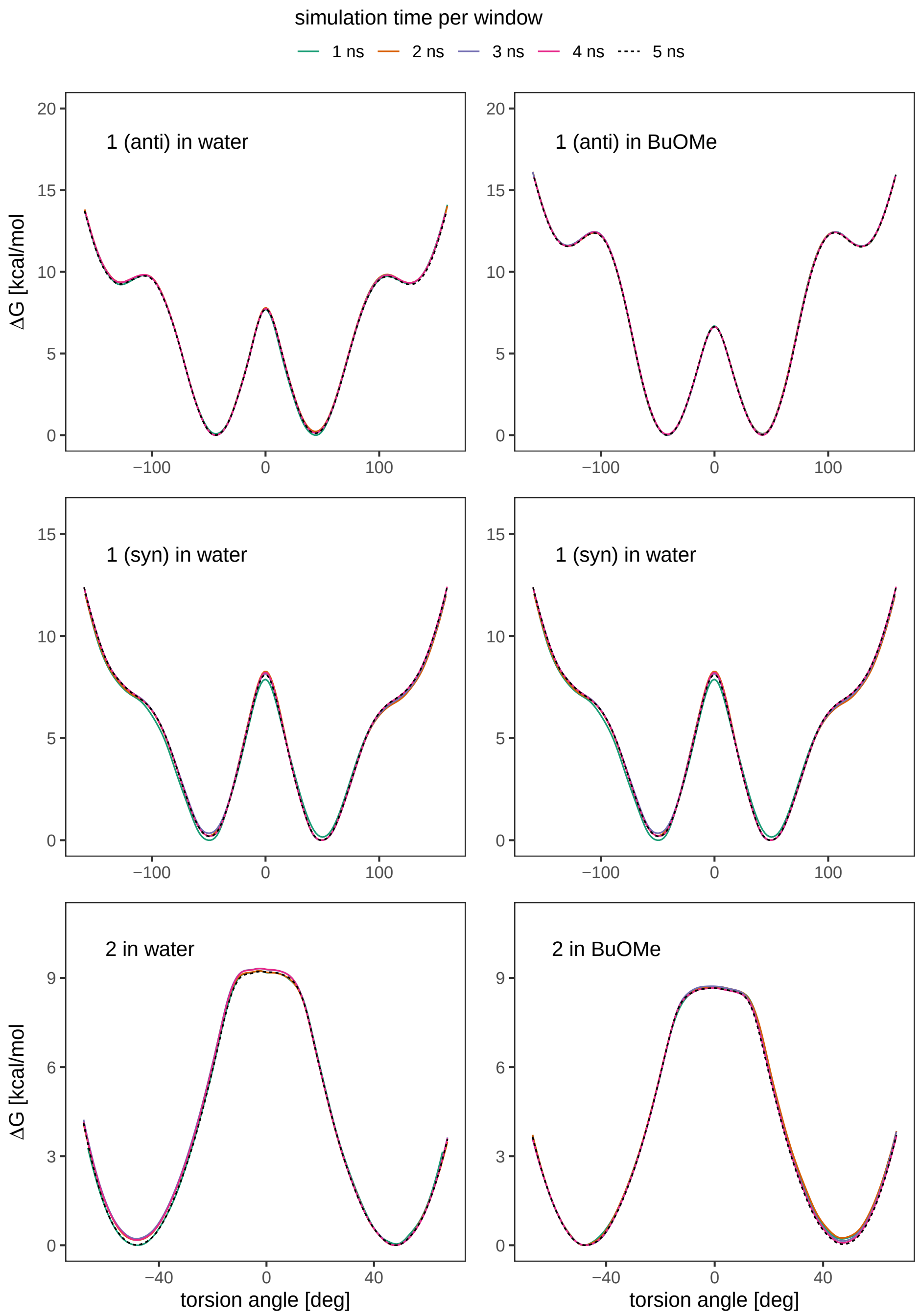

Figure **S4**: Free energy profiles computed with different simulation lengths. Profiles are cation 3 and **1** (*anti* charge distribution, top; and *syn* charge distribution, middle) and **2** (bottom).

5 Generation of data for *syn* and *anti* charge distribution of anion **1**

Figure S5 schematically illustrates how the force fields and the umbrella sampling data for the *syn* and *anti* conformation of anion **1** were generated.

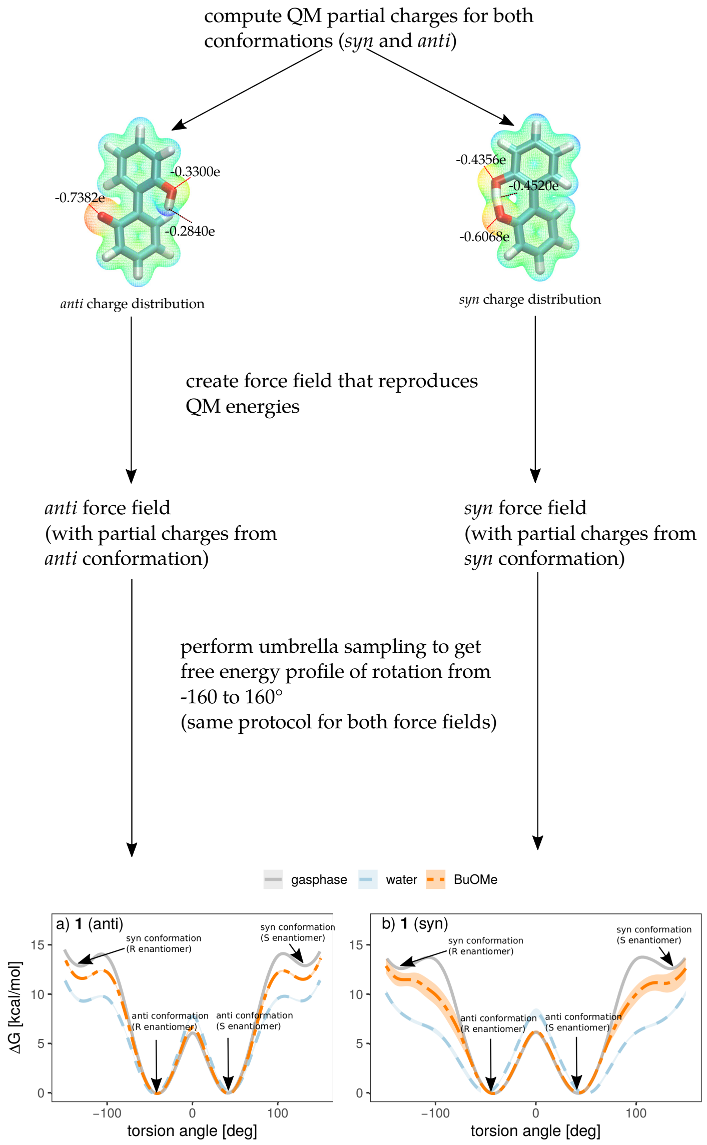

Figure **S5**: Workflow for generating data for both charge distributions.

6 Overview of simulations in solvent

cation	number of cations	anion	number of anions	solvent	number of solvent molecules	t/replica per window [ns]	total simulation time per window [ns]
3	1	1 (*anti*)	1	water (SWM4)	900	1	5
4	1	1 (*anti*)	1	water (SWM4)	900	1	5
5	1	1 (*anti*)	1	water (SWM4)	900	1	5
3	1	1 (*anti*)	1	n-butylmethylether (MBET)	160	1	5
4	1	1 (*anti*)	1	n-butylmethylether (MBET)	160	1	5
5	1	1 (*anti*)	1	n-butylmethylether (MBET)	160	1	5
3	1	1 (*anti*)	1	water (SWM4)	900	1	5
4	1	1 (*syn*)	1	water (SWM4)	900	1	5
5	1	1 (*syn*)	1	water (SWM4)	900	1	5
3	1	1 (*syn*)	1	n-butylmethylether (MBET)	160	1	5
4	1	1 (*syn*)	1	n-butylmethylether (MBET)	160	1	5
5	1	1 (*syn*)	1	1n-butylmethylether (MBET)	160	1	5
3	1	2	1	water (SWM4)	900	1	5
4	1	2	1	water (SWM4)	900	1	5
5	1	2	1	water (SWM4)	900	1	5
3	1	2	1	n-butylmethylether (MBET)	160	1	5
4	1	2	1	n-butylmethylether (MBET)	160	1	5
5	1	2	1	n-butylmethylether (MBET)	160	1	5

7 Hydrogen bonding of anion 2

Hydrogen bonds of anion **2**to water and counterions in BuOMe are depicted in Figures S6 and S7, respectively.

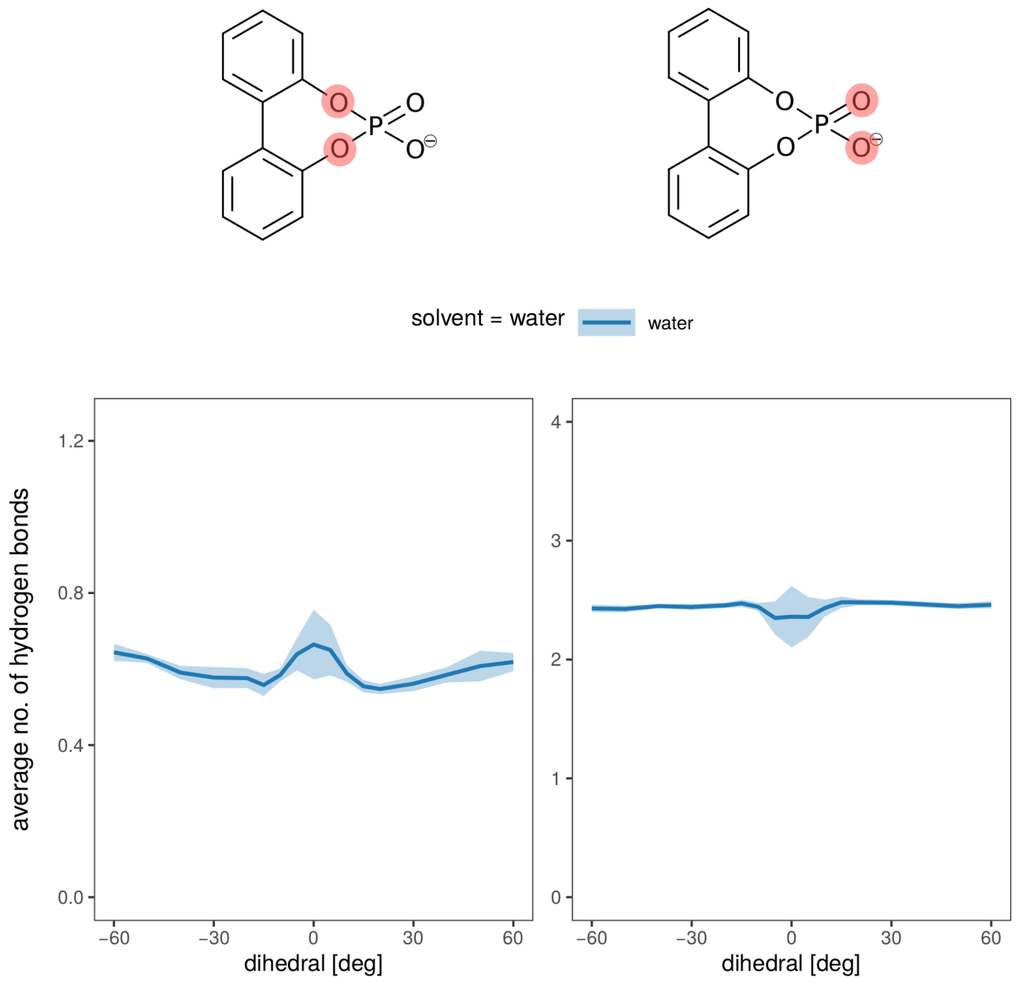

Figure **S6**: Hydrogen bonding between **2** and water (single ion pair of **3** and **2** in water).

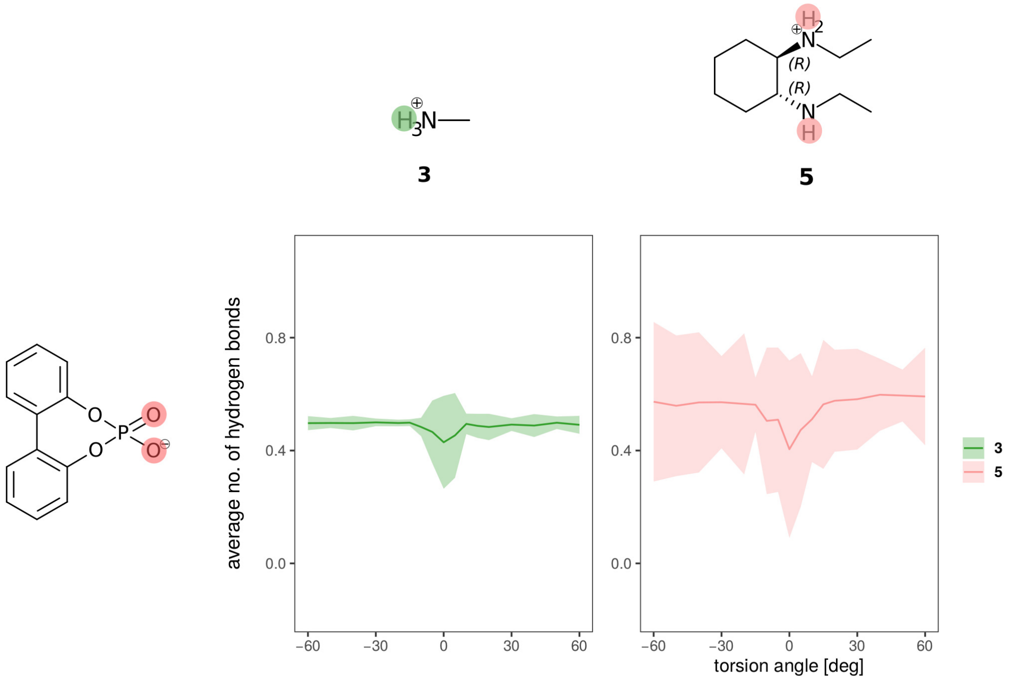

Figure **S7**: Hydrogen bonding between **2** and cations capable of hydrogen bonding, solvent=BuOMe.

8 Force Fields

Please note that in order to use the force fields, the 2018 version of the CHARMM Drude Force Field [2] (available from http://mackerell.umaryland.edu/charmm_drude_ff.shtml) needs to be loaded first, as parameters already available from the Drude Force Field are not included in the files below. Furthermore, two new atom types (CD2R6L and CD2R6M, same Lennard-Jones parameters as CD2R6A in the Drude Force Field) were defined to suitably model the biphenyl torsion. MASS statements and nonbonded parameters for these atom types need to be added to the master file of the CHARMM Drude Force Field to allow usage of the force fields listed below.

8.1 Cation 5

```
* Toppar stream file generated by
* hand from the CHARMM drude force field
*

read rtf card append
* Topologies for chiral ILs
*
41
AUTOGENERATE ANGLES DIHEDRALS DRUDE

RESI CHDA       1.000 ! param penalty=  39.900 ; charge penalty=  36.290
! initial charges + pols: RESP/RI-MP2/Sadlej//RI-MP2/6+31G(d), pols scaled by 0.85
! charges refitted to reproduce RI-MP2/cc-pVQZ//RI-MP2/6+31G(d) dipole moment
!  + interaction energies of hydrogen bonding sites with water
!
!       H8  H9
!         \/
!   H11   C2   H7  H20  H23 H26 H27   H33 H34
!     \ /   \ /     \(+)/    \ /       | /
!  H10-C1    C3------N19-----C21-------C28-H35
!      |      |
!  H15-C6    C4------N17-----C22-------C29-H30
!     / \    /\       |      / \        | \
!  H16    C5   H12   H18   H24 H25     H31 H32
!        / \
```

```
!       H13 H14
!
!
!
!
!
GROUP
ATOM C1     CD326A  -0.1902     ALPHA -1.400 THOLE 1.3
ATOM C2     CD326A   0.0987     ALPHA -1.337 THOLE 1.3
ATOM C3     CD316A  -0.2606     ALPHA -1.162 THOLE 1.3
ATOM C4     CD316A   0.2124     ALPHA -1.240 THOLE 1.3
ATOM C5     CD326A  -0.3085     ALPHA -1.399 THOLE 1.3
ATOM C6     CD326A   0.1423     ALPHA -1.377 THOLE 1.3
ATOM H7     HDA1A    0.1515
ATOM H8     HDA2A    0.0651
ATOM H9     HDA2A    0.0651
ATOM H10    HDA2A    0.0556
ATOM H11    HDA2A    0.0556
ATOM H12    HDA1A    0.0115
ATOM H13    HDA2A    0.1196
ATOM H14    HDA2A    0.1196
ATOM H15    HDA2A    0.0109
ATOM H16    HDA2A    0.0109
ATOM N17    ND3A2   -0.7274     ALPHA -1.208 THOLE 1.155
ATOM H18    HDP1A    0.3124
ATOM N19    ND3P2A  -0.3408     ALPHA -1.123 THOLE 1.3
ATOM H20    HDP1B    0.4052
ATOM C21    CD32A    0.1128     ALPHA -1.300 THOLE 1.005
ATOM C22    CD32A    0.2347     ALPHA -1.398 THOLE 1.005
ATOM H23    HDP1B    0.4052
ATOM H24    HDA2A    0.0435
ATOM H25    HDA2A    0.0435
ATOM H26    HDA2C    0.0952
ATOM H27    HDA2C    0.0952
ATOM C28    CD33A   -0.3156     ALPHA -1.560 THOLE 1.35
ATOM C29    CD33A   -0.3471     ALPHA -1.639 THOLE 1.35
ATOM H30    HDA3A    0.1106
ATOM H31    HDA3A    0.1106
ATOM H32    HDA3A    0.1106
ATOM H33    HDA3A    0.0973
ATOM H34    HDA3A    0.0973
ATOM H35    HDA3A    0.0973


BOND C1   C2
BOND C1   C6
BOND C1   H10
BOND C1   H11
BOND C2   C3
BOND C2   H8
BOND C2   H9
BOND C3   C4
BOND C3   H7
BOND C3   N19
BOND C4   C5
BOND C4   H12
BOND C4   N17
BOND C5   C6
BOND C5   H13
BOND C5   H14
BOND C6   H15
BOND C6   H16
BOND N17  H18
BOND N17  C22
BOND N19  H20
BOND N19  C21
BOND N19  H23
BOND C21  H26
BOND C21  H27
BOND C21  C28
BOND C22  H24
BOND C22  H25
BOND C22  C29
BOND C28  H33
BOND C28  H34
BOND C28  H35
BOND C29  H30
BOND C29  H31
BOND C29  H32

DONOR H20 N19
DONOR H23 N19
DONOR H18 N17

END

read param card append
```

```
* Parameters generated by analogy by
* CHARMM General Force Field (CGenFF) program version 2.2.0
*

! Penalties lower than 10 indicate the analogy is fair; penalties between 10
! and 50 mean some basic validation is recommended; penalties higher than
! 50 indicate poor analogy and mandate extensive validation/optimization.

BONDS
CD316A   CD316A   222.50      1.530 ! analogy, CHXM

CD316A   ND3P2A   260.70      1.5511 ! fitted
CD316A   ND3A2     298.75     1.4606 ! fitted

ANGLES

CD316A   CD316A   HDA1A     26.50    110.10   22.53   2.179  ! analogy, CHXM
ND3P2A   CD316A   HDA1A     34.50    110.10   22.53   2.179  ! analogy, CHXM
ND3A2    CD316A   HDA1A     34.50    110.10   22.53   2.179  ! analogy, CHXM

! nitrogen protons
CD316A   ND3A2    HDP1A      55.89    109.30 ! fitted
CD316A   ND3P2A   HDP1B      50.47    105.65 ! fitted

CD326A   CD316A   CD316A    58.35    113.60   11.16   2.561  ! analogy, CHXM

CD326A   CD316A   ND3A2      62.26    118.97 ! fitted
CD316A   CD316A   ND3A2      47.61    117.04 ! fitted


CD326A   CD316A   ND3P2A     91.62    111.50 ! fitted
CD316A   CD316A   ND3P2A     43.16    114.05 ! fitted


CD316A   ND3P2A   CD32A      45.55    115.20 ! fitted
CD316A   ND3A2    CD32A      49.88    111.22 ! fitted

DIHEDRALS

! ring pucker fit
CD326A   CD316A   CD316A   CD326A       1.4926  1     0.00 ! fitted
CD326A   CD316A   CD316A   CD326A       1.0708  2     0.00 ! fitted
CD326A   CD316A   CD316A   CD326A       0.0448  3     0.00 ! fitted
CD326A   CD316A   CD316A   CD326A       0.4577  4   180.00 ! fitted
CD326A   CD316A   CD316A   CD326A       0.9522  5     0.00 ! fitted
CD316A   CD316A   CD326A   CD326A       0.3376  1   180.00 ! fitted
CD316A   CD316A   CD326A   CD326A       0.3233  2   180.00 ! fitted
CD316A   CD316A   CD326A   CD326A       0.2660  3   180.00 ! fitted
CD316A   CD316A   CD326A   CD326A       0.1251  4     0.00 ! fitted
CD316A   CD316A   CD326A   CD326A       0.9243  5     0.00 ! fitted
ND3P2A   CD316A   CD326A   CD326A       1.0584  1     0.00 ! fitted
ND3P2A   CD316A   CD326A   CD326A       1.0356  2   180.00 ! fitted
ND3P2A   CD316A   CD326A   CD326A       0.5737  3     0.00 ! fitted
ND3P2A   CD316A   CD326A   CD326A       0.9663  4   180.00 ! fitted
ND3P2A   CD316A   CD326A   CD326A       0.4424  5     0.00 ! fitted
CD326A   CD316A   CD316A   ND3P2A       0.1507  1     0.00 ! fitted
CD326A   CD316A   CD316A   ND3P2A       0.2621  2   180.00 ! fitted
CD326A   CD316A   CD316A   ND3P2A       0.2711  3   180.00 ! fitted
CD326A   CD316A   CD316A   ND3P2A       0.9718  4     0.00 ! fitted
CD326A   CD316A   CD316A   ND3P2A       0.4276  5   180.00 ! fitted
CD326A   CD316A   CD316A   ND3A2        0.2405  1     0.00 ! fitted
CD326A   CD316A   CD316A   ND3A2        0.3538  2   180.00 ! fitted
CD326A   CD316A   CD316A   ND3A2        0.0376  3     0.00 ! fitted
CD326A   CD316A   CD316A   ND3A2        0.2326  4     0.00 ! fitted
CD326A   CD316A   CD316A   ND3A2        0.2790  5   180.00 ! fitted
ND3A2    CD316A   CD326A   CD326A       0.0522  1   180.00 ! fitted
ND3A2    CD316A   CD326A   CD326A       0.1446  2     0.00 ! fitted
ND3A2    CD316A   CD326A   CD326A       0.6116  3   180.00 ! fitted
ND3A2    CD316A   CD326A   CD326A       0.5823  4     0.00 ! fitted
ND3A2    CD316A   CD326A   CD326A       0.8877  5   180.00 ! fitted



! hydrogens
CD326A   CD316A   CD316A   HDA1A      0.190    3     0.00 ! analogy, CHXM
CD316A   CD316A   CD326A   HDA2A      0.190    3     0.00 ! analogy, CHEX
HDA2A    CD326A   CD316A   ND3P2A     0.190    3     0.00 ! analogy, CHXM
HDA1A    CD316A   CD316A   HDA1A      0.190    3     0.00 ! analogy, CHXM
HDA1A    CD316A   CD316A   ND3A2      0.190    3     0.00 ! analogy, CHXM
HDA1A    CD316A   CD316A   ND3P2A     0.190    3     0.00 ! analogy, CHXM
HDA2A    CD326A   CD316A   ND3A2      0.190    3     0.00 ! analogy, CHXM
ND3A2    CD316A   CD316A   ND3P2A     0.190    3     0.00 ! analogy, CHXM


!  C2-C3-N19-C21
CD326A   CD316A   ND3P2A   HDP1B        0.0335  3     0.00 ! fitted
CD326A   CD316A   ND3P2A   CD32A        0.1723  3   180.00 ! fitted
```

```
CD316A   CD316A   ND3P2A   HDP1B        0.3066  3     0.00 ! fitted
CD316A   CD316A   ND3P2A   CD32A        0.0646  3     0.00 ! fitted
HDA1A    CD316A   ND3P2A   HDP1B        0.1539  3     0.00 ! fitted
HDA1A    CD316A   ND3P2A   CD32A        0.1025  3   180.00 ! fitted


! C3-N19-C21-C28
HDA2C    CD32A    ND3P2A   CD316A       0.7100  1     0.00 ! fitted
HDA2C    CD32A    ND3P2A   CD316A       0.1769  3     0.00 ! fitted
CD33A    CD32A    ND3P2A   CD316A       0.7906  1   180.00 ! fitted
CD33A    CD32A    ND3P2A   CD316A       0.0297  3     0.00 ! fitted


! C5-C4-N17-C22
CD326A   CD316A   ND3A2    HDP1A        0.2729  3     0.00 ! fitted
CD326A   CD316A   ND3A2    CD32A        0.4293  3     0.00 ! fitted
CD316A   CD316A   ND3A2    HDP1A        1.3124  3   180.00 ! fitted
CD316A   CD316A   ND3A2    CD32A        0.8646  3   180.00 ! fitted
HDA1A    CD316A   ND3A2    HDP1A        0.7828  3     0.00 ! fitted
HDA1A    CD316A   ND3A2    CD32A        1.9053  3     0.00 ! fitted

! C4-N17-C22-C29
HDA2A    CD32A    ND3A2    CD316A       0.0108  3     0.00 ! fitted
CD33A    CD32A    ND3A2    CD316A       0.5756  3     0.00 ! fitted

IMPROPERS

END
RETURN
```

8.2 Anion 1, *anti* charge distribution

```
* Toppar stream file generated by
* hand from the CHARMM drude force field
*



read rtf card append
* Topologies for chiral ILs
*
41
AUTOGENERATE ANGLES DIHEDRALS DRUDE

RESI BDOA      -1.000 !
! initial charges + pols: RESP/RI-MP2/Sadlej//RI-MP2/6+31G(d), pols scaled by 0.724
! charges refitted to reproduce RI-MP2/cc-pVQZ//RI-MP2/6+31G(d) dipole moment
!  + interaction energies of hydrogen bonding sites with water
!
!
!
!         H14
!          |
!    H15  C4    H13
!      \ /  \  /
!       C5   C3
!       |    |
!       C6   C2
!      / \  /  \
!    H16  C1    O21--H22
!          |
!    O23  C7    H20
!     \  /  \  /
!      C8   C12
!       |    |
!      C9   C11
!      / \  /  \
!    H17  C10  H19
!          |
!         H18
!
!
GROUP
ATOM C1   CD2R6L -0.1474  ALPHA -1.334  THOLE 1.21
ATOM C2   CD2R6M  0.3169  ALPHA -1.101  THOLE 1.26
ATOM C3   CD2R6A -0.2476  ALPHA -1.390  THOLE 1.26
ATOM C4   CD2R6A -0.1916  ALPHA -1.441  THOLE 1.26
ATOM C5   CD2R6A -0.1710  ALPHA -1.469  THOLE 1.26
ATOM C6   CD2R6A -0.1267  ALPHA -1.235  THOLE 1.26
ATOM C7   CD2R6L -0.1325  ALPHA -1.474  THOLE 1.14
ATOM C8   CD2R6I  0.5015  ALPHA -1.285  THOLE 1.19
ATOM C9   CD2R6A -0.3774  ALPHA -1.548  THOLE 1.384
ATOM C10  CD2R6A -0.1078  ALPHA -1.522  THOLE 1.384
ATOM C11  CD2R6A -0.2963  ALPHA -1.740  THOLE 1.34
ATOM C12  CD2R6A -0.1019  ALPHA -1.369  THOLE 1.35
ATOM H13  HDR6A   0.1327
ATOM H14  HDR6A   0.1092
ATOM H15  HDR6A   0.1155
ATOM H16  HDR6A   0.1312
ATOM H17  HDR6A   0.1057
ATOM H18  HDR6A   0.0764
ATOM H19  HDR6A   0.0939
ATOM H20  HDR6A   0.1014
ATOM O21  OD31C  -0.0000  ALPHA -0.798  THOLE 1.1 !
ATOM H22  HDP1A   0.2840
ATOM O23  OD30E  -0.7382  ALPHA -1.032  THOLE 1.13 !
ATOM LP1A LPD    -0.1650
ATOM LP1B LPD    -0.1650

BOND C1   C2
BOND C1   C6
BOND C1   C7
BOND C2   C3
BOND C2   O21
BOND C3   C4
BOND C3   H13
BOND C4   C5
BOND C4   H14
BOND C5   C6
BOND C5   H15
BOND C6   H16
BOND C7   C8
BOND C7   C12
BOND C8   C9
BOND C8   O23
BOND C9   C10
BOND C9   H17
```

```
BOND C10  C11
BOND C10  H18
BOND C11  C12
BOND C11  H19
BOND C12  H20
BOND O21  H22
BOND O21 LP1A
BOND O21 LP1B

LONEPAIR relative LP1A O21 C2 H22 distance 0.35 angle 110.9 dihe  91.0
LONEPAIR relative LP1B O21 C2 H22 distance 0.35 angle 110.9 dihe 269.0
ANISOTROPY  O21 C2 LP1A LP1B A11 0.8108 A22 1.2162

ACCEPTOR O23
ACCEPTOR O21
DONOR H22 O21

END

read param card append

BONDS
CD2R6A   CD2R6L   305.00      1.375 ! analogy, BENZ
CD2R6I   CD2R6L   305.00      1.375 ! analogy, BENZ
CD2R6M   CD2R6L   305.00      1.375 ! analogy, BENZ
CD2R6M   CD2R6A   305.00      1.375 ! analogy, BENZ
CD2R6L   CD2R6L   368.41      1.4590 ! fitted
OD31C    CD2R6M   334.30      1.411 ! analogy, PHEN

ANGLES
CD2R6M   CD2R6L   CD2R6A    40.00    120.00   35.00   2.4162  ! analogy, BENZ
CD2R6L   CD2R6A   CD2R6A    40.00    120.00   35.00   2.4162  ! analogy, BENZ
CD2R6L   CD2R6A   HDR6A     30.00    120.00   22.00   2.1525  ! analogy, BENZ
CD2R6M   CD2R6A   HDR6A     30.00    120.00   22.00   2.1525  ! analogy, BENZ
CD2R6L   CD2R6I   CD2R6A    40.00    120.00   35.00   2.4162  ! analogy, BENZ
CD2R6L   CD2R6M   CD2R6A    40.00    120.00   35.00   2.4162  ! analogy, BENZ
CD2R6I   CD2R6L   CD2R6A    40.00    120.00   35.00   2.4162  ! analogy, BENZ
CD2R6L   CD2R6I   OD30E     55.20    127.80 ! analogy, PHET
CD2R6A   CD2R6L   CD2R6L    45.80    122.30 ! analogy, TOLU
CD2R6I   CD2R6L   CD2R6L    45.80    122.30 ! analogy, TOLU
CD2R6M   CD2R6L   CD2R6L    45.80    122.30 ! analogy, TOLU
OD31C    CD2R6M   CD2R6A    45.20    120.00 ! analogy, PHEN
OD31C    CD2R6M   CD2R6L    45.20    120.00 ! analogy, PHEN
CD2R6A   CD2R6A   CD2R6M    50.00    118.20 ! analogy, PHET
CD2R6M   OD31C    HDP1A     65.00    108.00 ! analogy, PHEN

DIHEDRALS

!aromatics
CD2R6A   CD2R6A   CD2R6A   CD2R6L      2.800    2   180.00 ! analogy, BENZ
CD2R6A   CD2R6A   CD2R6A   CD2R6M      2.800    2   180.00 ! analogy, BENZ
CD2R6A   CD2R6A   CD2R6M   CD2R6L      2.800    2   180.00 ! analogy, BENZ
CD2R6A   CD2R6A   CD2R6I   CD2R6L      2.800    2   180.00 ! analogy, BENZ

CD2R6A   CD2R6A   CD2R6L   CD2R6A      2.800    2   180.00 ! analogy, BENZ
CD2R6I   CD2R6L   CD2R6A   CD2R6A      2.800    2   180.00 ! analogy, PHET
CD2R6M   CD2R6L   CD2R6A   CD2R6A      2.800    2   180.00 ! analogy, PHET
CD2R6A   CD2R6I   CD2R6L   CD2R6A      2.800    2   180.00 ! analogy, PHET
CD2R6A   CD2R6M   CD2R6L   CD2R6A      2.800    2   180.00 ! analogy, PHET

!aromatic H
CD2R6A   CD2R6L   CD2R6A   HDR6A       4.200    2   180.00 ! analogy, BENZ
CD2R6I   CD2R6L   CD2R6A   HDR6A       4.200    2   180.00 ! analogy, BENZ
CD2R6M   CD2R6L   CD2R6A   HDR6A       4.200    2   180.00 ! analogy, BENZ
CD2R6L   CD2R6I   CD2R6A   HDR6A       4.200    2   180.00 ! analogy, BENZ
CD2R6L   CD2R6A   CD2R6A   HDR6A       4.200    2   180.00 ! analogy, BENZ
CD2R6M   CD2R6A   CD2R6A   HDR6A       4.200    2   180.00 ! analogy, BENZ
CD2R6L   CD2R6L   CD2R6A   HDR6A       4.200    2   180.00 ! analogy, BENZ
CD2R6L   CD2R6M   CD2R6A   HDR6A       4.200    2   180.00 ! analogy, BENZ
OD31C    CD2R6M   CD2R6A   HDR6A       4.200    2   180.00 ! analogy, PHEN

! out-of-plane-dihedrals
CD2R6L   CD2R6L   CD2R6M   CD2R6A       2.3616  2   180.00 ! fitted
CD2R6A   CD2R6A   CD2R6L   CD2R6L       1.8974  2   180.00 ! fitted
CD2R6A   CD2R6I   CD2R6L   CD2R6L       1.2170  2   180.00 ! fitted



! aryl-aryl rotation dihedrals
CD2R6A   CD2R6L   CD2R6L   CD2R6A       1.6107  1   180.00 ! fitted
CD2R6A   CD2R6L   CD2R6L   CD2R6A       1.3013  2   180.00 ! fitted
CD2R6A   CD2R6L   CD2R6L   CD2R6A       0.7234  3     0.00 ! fitted
CD2R6A   CD2R6L   CD2R6L   CD2R6A       0.1091  4   180.00 ! fitted
CD2R6A   CD2R6L   CD2R6L   CD2R6I       1.7279  1     0.00 ! fitted
CD2R6A   CD2R6L   CD2R6L   CD2R6I       0.9956  2   180.00 ! fitted
CD2R6A   CD2R6L   CD2R6L   CD2R6I       0.0894  3     0.00 ! fitted
CD2R6A   CD2R6L   CD2R6L   CD2R6I       0.6154  4     0.00 ! fitted
```

```
CD2R6A   CD2R6L   CD2R6L   CD2R6M       1.6409  1     0.00 ! fitted
CD2R6A   CD2R6L   CD2R6L   CD2R6M       0.9894  2   180.00 ! fitted
CD2R6A   CD2R6L   CD2R6L   CD2R6M       0.4753  3   180.00 ! fitted
CD2R6A   CD2R6L   CD2R6L   CD2R6M       0.2063  4     0.00 ! fitted
CD2R6I   CD2R6L   CD2R6L   CD2R6M       1.7374  1   180.00 ! fitted
CD2R6I   CD2R6L   CD2R6L   CD2R6M       0.7900  2   180.00 ! fitted
CD2R6I   CD2R6L   CD2R6L   CD2R6M       0.2783  3   180.00 ! fitted
CD2R6I   CD2R6L   CD2R6L   CD2R6M       1.0556  4     0.00 ! fitted
CD2R6L   CD2R6M   OD31C    HDP1A        0.3275  1   180.00 ! fitted
CD2R6L   CD2R6M   OD31C    HDP1A        0.5615  2   180.00 ! fitted
CD2R6L   CD2R6M   OD31C    HDP1A        0.3067  3   180.00 ! fitted
CD2R6L   CD2R6M   OD31C    HDP1A        0.5427  4   180.00 ! fitted
CD2R6A   CD2R6M   OD31C    HDP1A        0.2461  1     0.00 ! fitted
CD2R6A   CD2R6M   OD31C    HDP1A        0.1388  2   180.00 ! fitted
CD2R6A   CD2R6M   OD31C    HDP1A        0.0414  3     0.00 ! fitted
CD2R6A   CD2R6M   OD31C    HDP1A        0.3533  4   180.00 ! fitted

! O-C-Cbip-C
OD31C    CD2R6M   CD2R6L   CD2R6A      3.100    2   180.00 ! analogy, PHEN
OD31C    CD2R6M   CD2R6L   CD2R6L      3.100    2   180.00 ! analogy, PHEN

OD31C    CD2R6M   CD2R6A   CD2R6A      3.100    2   180.00 ! analogy, PHEN

OD30E   CD2R6I   CD2R6L   CD2R6A       3.100    2   180.00 ! analogy, PHET
OD30E    CD2R6I   CD2R6L   CD2R6L      3.100    2   180.00 ! analogy, PHEN

IMPROPERS
CD2R6L   CD2R6A   CD2R6L   CD2R6M       50.000   0     0.00
CD2R6L   CD2R6A   CD2R6L   CD2R6I       50.000   0     0.00


END
RETURN
```

8.3 Anion 1, *syn* charge distribution

```
* Toppar stream file generated by
* hand from the CHARMM drude force field
*



read rtf card append
* Topologies for chiral ILs
*
41
AUTOGENERATE ANGLES DIHEDRALS DRUDE

RESI BDOA      -1.000 !
! initial charges + pols: RESP/RI-MP2/Sadlej//RI-MP2/6+31G(d), pols scaled by 0.724
! charges refitted to reproduce RI-MP2/cc-pVQZ//RI-MP2/6+31G(d) dipole moment
!  + interaction energies of hydrogen bonding sites with water
!
!
!
!         H14
!          |
!    H15  C4    H13
!      \ /  \  /
!       C5   C3
!       |    |
!       C6   C2
!     /  \  /  \
!  H16    C1   O21--H21
!         |  LP1A LP1B
!        |
!  H20    C7    O23
!     \  /  \  /
!      C12   C8
!       |    |
!      C11   C9
!      / \  /  \
!    H19  C10  H17
!          |
!         H18
GROUP
ATOM C1   CD2R6L -0.1546  ALPHA -1.269  THOLE 1.1
ATOM C2   CD2R6M  0.3298  ALPHA -1.321  THOLE 1.27
ATOM C3   CD2R6A -0.2820  ALPHA -1.401  THOLE 1.27
ATOM C4   CD2R6A -0.2342  ALPHA -1.467  THOLE 1.27
ATOM C5   CD2R6A -0.1020  ALPHA -1.535  THOLE 1.27
ATOM C6   CD2R6A -0.1043  ALPHA -1.337  THOLE 1.27
ATOM C7   CD2R6L -0.1525  ALPHA -1.526  THOLE 1.12
ATOM C8   CD2R6I  0.4534  ALPHA -1.177  THOLE 1.19
ATOM C9   CD2R6A -0.4714  ALPHA -1.475  THOLE 1.394
ATOM C10  CD2R6A -0.2571  ALPHA -1.499  THOLE 1.394
ATOM C11  CD2R6A -0.1402  ALPHA -1.650  THOLE 1.33
ATOM C12  CD2R6A -0.1406  ALPHA -1.387  THOLE 1.394
ATOM H13  HDR6A   0.1046
ATOM H14  HDR6A   0.1011
ATOM H15  HDR6A   0.1011
ATOM H16  HDR6A   0.1062
ATOM H17  HDR6A   0.1087
ATOM H18  HDR6A   0.1135
ATOM H19  HDR6A   0.1116
ATOM H20  HDR6A   0.1003
ATOM O21  OD31C  -0.0000  ALPHA -0.765  THOLE 1.1
ATOM H22  HDP1A   0.4520
ATOM O23  OD30E  -0.6068  ALPHA -0.862  THOLE 1.13
ATOM LP1A LPD    -0.2183
ATOM LP1B LPD    -0.2183

BOND C1   C2
BOND C1   C6
BOND C1   C7
BOND C2   C3
BOND C2   O21
BOND C3   C4
BOND C3   H13
BOND C4   C5
BOND C4   H14
BOND C5   C6
BOND C5   H15
BOND C6   H16
BOND C7   C8
BOND C7   C12
BOND C8   C9
BOND C8   O23
BOND C9   C10
BOND C9   H17
BOND C10  C11
```

```
BOND C10  H18
BOND C11  C12
BOND C11  H19
BOND C12  H20
BOND O21  H22
BOND O21 LP1A
BOND O21 LP1B

LONEPAIR relative LP1A O21 C2 H22 distance 0.35 angle 110.9 dihe  91.0
LONEPAIR relative LP1B O21 C2 H22 distance 0.35 angle 110.9 dihe 269.0
ANISOTROPY  O21 C2 LP1A LP1B A11 0.8108 A22 1.2162

ACCEPTOR O23
ACCEPTOR O21
DONOR H22 O21


END

read param card append



BONDS
CD2R6A   CD2R6L   305.00      1.375 ! analogy, BENZ
CD2R6I   CD2R6L   305.00      1.375 ! analogy, BENZ
CD2R6M   CD2R6L   305.00      1.375 ! analogy, BENZ
CD2R6M   CD2R6A   305.00      1.375 ! analogy, BENZ
CD2R6L   CD2R6L    359.94     1.448 ! fitted
OD31C    CD2R6M   334.30      1.411 ! analogy, PHEN

ANGLES
CD2R6M   CD2R6L   CD2R6A    40.00    120.00   35.00   2.4162  ! analogy, BENZ
CD2R6L   CD2R6A   CD2R6A    40.00    120.00   35.00   2.4162  ! analogy, BENZ
CD2R6L   CD2R6A   HDR6A     30.00    120.00   22.00   2.1525  ! analogy, BENZ
CD2R6M   CD2R6A   HDR6A     30.00    120.00   22.00   2.1525  ! analogy, BENZ
CD2R6L   CD2R6I   CD2R6A    40.00    120.00   35.00   2.4162  ! analogy, BENZ
CD2R6L   CD2R6M   CD2R6A    40.00    120.00   35.00   2.4162  ! analogy, BENZ
CD2R6I   CD2R6L   CD2R6A    40.00    120.00   35.00   2.4162  ! analogy, BENZ
CD2R6L   CD2R6I   OD30E     55.20    127.80 ! analogy, PHET
CD2R6A   CD2R6L   CD2R6L    45.80    122.30 ! analogy, TOLU
CD2R6I   CD2R6L   CD2R6L    45.80    122.30 ! analogy, TOLU
CD2R6M   CD2R6L   CD2R6L    45.80    122.30 ! analogy, TOLU
OD31C    CD2R6M   CD2R6A    45.20    120.00 ! analogy, PHEN
OD31C    CD2R6M   CD2R6L    45.20    120.00 ! analogy, PHEN
CD2R6A   CD2R6A   CD2R6M    50.00    118.20 ! analogy, PHEN
CD2R6M   OD31C    HDP1A     65.00    108.00 ! analogy, PHEN

DIHEDRALS

!aromatics
CD2R6A   CD2R6A   CD2R6A   CD2R6L      2.800    2   180.00 ! analogy, BENZ
CD2R6A   CD2R6A   CD2R6A   CD2R6M      2.800    2   180.00 ! analogy, BENZ
CD2R6A   CD2R6A   CD2R6M   CD2R6L      2.800    2   180.00 ! analogy, BENZ
CD2R6A   CD2R6A   CD2R6I   CD2R6L      2.800    2   180.00 ! analogy, BENZ

CD2R6A   CD2R6A   CD2R6L   CD2R6A      2.800    2   180.00 ! analogy, BENZ
CD2R6I   CD2R6L   CD2R6A   CD2R6A      2.800    2   180.00 ! analogy, BENZ
CD2R6M   CD2R6L   CD2R6A   CD2R6A      2.800    2   180.00 ! analogy, BENZ
CD2R6A   CD2R6I   CD2R6L   CD2R6A      2.800    2   180.00 ! analogy, BENZ
CD2R6A   CD2R6M   CD2R6L   CD2R6A      2.800    2   180.00 ! analogy, BENZ

!aromatic H
CD2R6A   CD2R6L   CD2R6A   HDR6A       4.200    2   180.00 !  analogy, BENZ
CD2R6I   CD2R6L   CD2R6A   HDR6A       4.200    2   180.00 !  analogy, BENZ
CD2R6M   CD2R6L   CD2R6A   HDR6A       4.200    2   180.00 !  analogy, BENZ
CD2R6L   CD2R6I   CD2R6A   HDR6A       4.200    2   180.00 !  analogy, BENZ
CD2R6L   CD2R6A   CD2R6A   HDR6A       4.200    2   180.00 !  analogy, BENZ
CD2R6M   CD2R6A   CD2R6A   HDR6A       4.200    2   180.00 !  analogy, BENZ
CD2R6L   CD2R6L   CD2R6A   HDR6A       4.200    2   180.00 !  analogy, TOLU
CD2R6L   CD2R6M   CD2R6A   HDR6A       4.200    2   180.00 !  analogy, BENZ
OD31C    CD2R6M   CD2R6A   HDR6A       4.200    2   180.00 !  analogy, PHEN

! out-of-plane-dihedrals
CD2R6L   CD2R6L   CD2R6M   CD2R6A       1.5616  2   180.00 ! fitted
CD2R6A   CD2R6A   CD2R6L   CD2R6L       1.1974  2   180.00 ! fitted
CD2R6A   CD2R6I   CD2R6L   CD2R6L       0.1170  2   180.00 ! fitted

! aryl-aryl rotation dihedrals
CD2R6A   CD2R6L   CD2R6L   CD2R6A       1.3492  1   180.00 ! fitted
CD2R6A   CD2R6L   CD2R6L   CD2R6A       1.2255  2   180.00 ! fitted
CD2R6A   CD2R6L   CD2R6L   CD2R6A       0.9003  3     0.00 ! fitted
CD2R6A   CD2R6L   CD2R6L   CD2R6A       0.3422  4   180.00 ! fitted
CD2R6A   CD2R6L   CD2R6L   CD2R6I       1.5081  1     0.00 ! fitted
CD2R6A   CD2R6L   CD2R6L   CD2R6I       0.7798  2   180.00 ! fitted
CD2R6A   CD2R6L   CD2R6L   CD2R6I       0.0439  3   180.00 ! fitted
CD2R6A   CD2R6L   CD2R6L   CD2R6I       0.5471  4     0.00 ! fitted
```

```
CD2R6A   CD2R6L   CD2R6L   CD2R6M       1.3829  1     0.00 ! fitted
CD2R6A   CD2R6L   CD2R6L   CD2R6M       0.8615  2   180.00 ! fitted
CD2R6A   CD2R6L   CD2R6L   CD2R6M       0.6010  3   180.00 ! fitted
CD2R6A   CD2R6L   CD2R6L   CD2R6M       0.0722  4   180.00 ! fitted
CD2R6I   CD2R6L   CD2R6L   CD2R6M       1.5139  1   180.00 ! fitted
CD2R6I   CD2R6L   CD2R6L   CD2R6M       0.5625  2   180.00 ! fitted
CD2R6I   CD2R6L   CD2R6L   CD2R6M       0.1961  3   180.00 ! fitted
CD2R6I   CD2R6L   CD2R6L   CD2R6M       1.0337  4     0.00 ! fitted
CD2R6L   CD2R6M   OD31C    HDP1A        0.2165  1     0.00 ! fitted
CD2R6L   CD2R6M   OD31C    HDP1A        0.8886  2   180.00 ! fitted
CD2R6L   CD2R6M   OD31C    HDP1A        0.1292  3   180.00 ! fitted
CD2R6L   CD2R6M   OD31C    HDP1A        0.8718  4   180.00 ! fitted
CD2R6A   CD2R6M   OD31C    HDP1A        0.3594  1   180.00 ! fitted
CD2R6A   CD2R6M   OD31C    HDP1A        0.0985  2   180.00 ! fitted
CD2R6A   CD2R6M   OD31C    HDP1A        0.3162  3   180.00 ! fitted
CD2R6A   CD2R6M   OD31C    HDP1A        0.4175  4   180.00 ! fitted


OD31C    CD2R6M   CD2R6L   CD2R6A      3.100    2   180.00 ! analogy, PHEN
OD31C    CD2R6M   CD2R6L   CD2R6L      3.100    2   180.00 ! analogy, PHEN

OD31C    CD2R6M   CD2R6A   CD2R6A      3.100    2   180.00 ! analogy, PHEN

OD30E   CD2R6I   CD2R6L   CD2R6A       3.100    2   180.00 ! analogy, PHET
OD30E    CD2R6I   CD2R6L   CD2R6L      3.100    2   180.00 ! analogy, PHEN

IMPROPERS
CD2R6L   CD2R6A   CD2R6L   CD2R6M       50.000   0     0.00
CD2R6L   CD2R6A   CD2R6L   CD2R6I       50.000   0     0.00


END
RETURN
```

8.4 Anion 2

```
* Toppar stream file generated by
* hand from the CHARMM drude force field
*

read rtf card append
* Topologies for chiral ILs
*
41
AUTOGENERATE ANGLES DIHEDRALS DRUDE

RESI AN4      -1.000 !
! initial charges + pols: RESP/RI-MP2/Sadlej//RI-MP2/6+31G(d), pols scaled by 0.6
! charges refitted to reproduce RI-MP2/cc-pVQZ//RI-MP2/6+31G(d) dipole moment
!  + interaction energies of hydrogen bonding sites with water
!
!
!          H13
!           |
!     H15   C9    H10
!       \ /    \ /
!        C12    C7
!        |      |
!        C11    C6
!       / \    /  \
!      H14  C8     O2   O4
!           |       \  /
!           |        P1
!           |       / \
!      H24 C18    O3   O5
!       \ /   \  /
!       C21   C16
!        |     |
!       C22   C17
!       / \   / \
!     H25  C19   H20
!           |
!          H23
!
GROUP
ATOM P1    PD1AN    1.1364  ALPHA  -0.921 THOLE 2.2
ATOM O2    OD30BN  -0.4857  ALPHA  -0.742 THOLE 0.6
ATOM O3    OD30BN  -0.4857  ALPHA  -0.742 THOLE 0.6
ATOM O4    OD2C2C -0.6943   ALPHA  -0.798  THOLE 1.2
ATOM O5    OD2C2C -0.6943   ALPHA  -0.798  THOLE 1.2
ATOM C6    CD2R6M  0.3503   ALPHA  -0.845  THOLE 1.1
ATOM C7    CD2R6A -0.1953   ALPHA  -1.071  THOLE 1.47
ATOM C8    CD2R6L -0.0495   ALPHA  -0.946  THOLE 1.05
ATOM C9    CD2R6A -0.1108   ALPHA  -1.139  THOLE 1.45
ATOM H10   HDR6A   0.0707
ATOM C11   CD2R6A -0.1019   ALPHA  -1.082  THOLE 1.4
ATOM C12   CD2R6A -0.1569   ALPHA  -1.173  THOLE 1.3
ATOM H13   HDR6A   0.1031
ATOM H14   HDR6A   0.0940
ATOM H15   HDR6A   0.1081
ATOM C16   CD2R6M  0.3503   ALPHA  -0.845  THOLE 1.1
ATOM C17   CD2R6A -0.1953   ALPHA  -1.071  THOLE 1.47
ATOM C18   CD2R6L -0.0495   ALPHA  -0.946  THOLE 1.05
ATOM C19   CD2R6A -0.1108   ALPHA  -1.139  THOLE 1.45
ATOM H20   HDR6A   0.0707
ATOM C21   CD2R6A -0.1019   ALPHA  -1.082  THOLE 1.4
ATOM C22   CD2R6A -0.1569   ALPHA  -1.173  THOLE 1.3
ATOM H23   HDR6A   0.1031
ATOM H24   HDR6A   0.0940
ATOM H25   HDR6A   0.1081
IMPR C8 C11 C18 C6
IMPR C18 C21 C8 C16
ACCEPTOR O2
ACCEPTOR O3
ACCEPTOR O4
ACCEPTOR O5

BOND P1   O2
BOND P1   O3
BOND P1   O4
BOND P1   O5
BOND O2   C6
BOND O3   C16
BOND C6   C7
BOND C6   C8
BOND C7   C9
BOND C7   H10
BOND C8   C11
BOND C8   C18
BOND C9   C12
BOND C9   H13
```

```
BOND C11  C12
BOND C11  H14
BOND C12  H15
BOND C16  C17
BOND C16  C18
BOND C17  C19
BOND C17  H20
BOND C18  C21
BOND C19  C22
BOND C19  H23
BOND C21  C22
BOND C21  H24
BOND C22  H25
!RING planar 6 C8 C6 C7 C9 C12 C11
!RING planar 6 C18 C16 C17 C19 C22 C12
!comment nonplanar 7 P1 O2 C6 C8 C18 C16 O3

ANISOTROPY O2 O4 O3 O5 A11 1.0000  A22 0.6000
ANISOTROPY O3 O5 O2 O4 A11 1.0000  A22 0.6000
ANISOTROPY O4 P1  O2 O3 A11 0.6000  A22 1.0000
ANISOTROPY O5 P1  O3 O2 A11 0.6000  A22 1.0000

IC O3  O2  *P1  O4        0.0000    0.00  120.00    0.00   0.0000
IC O3  O2  *P1  O5        0.0000    0.00 -120.00    0.00   0.0000
IC O3  P1  O2   C6        0.0000    0.00  180.00    0.00   0.0000
IC P1  O2  C6   C8        0.0000    0.00  180.00    0.00   0.0000
IC C8  O2  *C6  C7        0.0000    0.00  180.00    0.00   0.0000
IC O2  C6  C7   C9        0.0000    0.00  180.00    0.00   0.0000
IC C9  C6  *C7  H10       0.0000    0.00  180.00    0.00   0.0000
IC O2  C6  C8   C18       0.0000    0.00  180.00    0.00   0.0000
IC C18 C6  *C8  C11       0.0000    0.00  180.00    0.00   0.0000
IC C6  C7  C9   C12       0.0000    0.00  180.00    0.00   0.0000
IC C12 C7  *C9  H13       0.0000    0.00  180.00    0.00   0.0000
IC C12 C8  *C11 H14       0.0000    0.00  180.00    0.00   0.0000
IC C11 C9  *C12 H15       0.0000    0.00  180.00    0.00   0.0000
IC O2  P1  O3   C16       0.0000    0.00  180.00    0.00   0.0000
IC C18 O3  *C16 C17       0.0000    0.00  180.00    0.00   0.0000
IC O3  C16 C17  C19       0.0000    0.00  180.00    0.00   0.0000
IC C19 C16 *C17 H20       0.0000    0.00  180.00    0.00   0.0000
IC C16 C8  *C18 C21       0.0000    0.00  180.00    0.00   0.0000
IC C16 C17 C19  C22       0.0000    0.00  180.00    0.00   0.0000
IC C22 C17 *C19 H23       0.0000    0.00  180.00    0.00   0.0000
IC C22 C18 *C21 H24       0.0000    0.00  180.00    0.00   0.0000
IC C21 C19 *C22 H25       0.0000    0.00  180.00    0.00   0.0000


END

read param card append
* parameters generated according to protocol for CHARMM Drude FF
*

! Penalties lower than 10 indicate the analogy is fair; penalties between 10
! and 50 mean some basic validation is recommended; penalties higher than
! 50 indicate poor analogy and mandate extensive validation/optimization.

BONDS
OD30BN    CD2R6M    335.00     1.420 ! analogy, BENZ

CD2R6M   CD2R6A    305.00      1.375 ! analogy, BENZ
CD2R6M   CD2R6L    305.00      1.375 ! analogy, BENZ
CD2R6L   CD2R6A    305.00      1.375 ! analogy, BENZ

CD2R6L   CD2R6L    315.28     1.4450 ! fitted


ANGLES
PD1AN     OD30BN    CD2R6M 40.00    112.50 ! analogy, DMP
OD30BN    CD2R6M   CD2R6A 55.20    127.80 ! analogy, PHET
OD30BN    CD2R6M   CD2R6L 55.20    127.80 ! analogy, PHET

CD2R6A   CD2R6M   CD2R6L 40.00    120.00   35.00   2.4162  ! analogy, BENZ
CD2R6M   CD2R6A   CD2R6A 40.00    120.00   35.00   2.4162  ! analogy, BENZ
CD2R6L   CD2R6A   CD2R6A 40.00    120.00   35.00   2.4162  ! analogy, BENZ

CD2R6M   CD2R6A   HDR6A 30.00    120.00   22.00   2.1525  ! analogy, BENZ
CD2R6L   CD2R6A   HDR6A  30.00    120.00   22.00   2.1525  ! analogy, BENZ

CD2R6A   CD2R6L   CD2R6M     27.32    113.97 ! fitted
CD2R6L   CD2R6L   CD2R6M     50.20    120.94 ! fitted
CD2R6A   CD2R6L   CD2R6L     40.93    123.60 ! fitted



DIHEDRALS
```

```
OD30BN    CD2R6M   CD2R6A   CD2R6A 3.100    2   180.00 ! analogy, PHEN
OD30BN    CD2R6M   CD2R6L   CD2R6A  3.100    2   180.00 ! analogy, PHEN
OD30BN    CD2R6M   CD2R6L   CD2R6L 3.100    2   180.00 ! analogy, PHEN

CD2R6M   CD2R6A   CD2R6A   CD2R6A  2.800    2   180.00 ! analogy, BENZ
CD2R6M   CD2R6L   CD2R6A   CD2R6A 2.800    2   180.00 ! analogy, BENZ
CD2R6A   CD2R6M   CD2R6L   CD2R6A  2.800    2   180.00 ! analogy, BENZ
CD2R6L   CD2R6M   CD2R6A   CD2R6A 2.800    2   180.00 ! analogy, BENZ
CD2R6L   CD2R6A   CD2R6A   CD2R6A  2.800    2   180.00 ! analogy, BENZ

OD30BN    CD2R6M   CD2R6A   HDR6A  4.200    2   180.00 ! analogy, PHEN
CD2R6M   CD2R6A   CD2R6A   HDR6A 4.200    2   180.00 ! analogy, BENZ
CD2R6M   CD2R6L   CD2R6A   HDR6A 4.200    2   180.00 ! analogy, BENZ
CD2R6L   CD2R6M   CD2R6A   HDR6A  4.200    2   180.00 ! analogy, BENZ
CD2R6L   CD2R6A   CD2R6A   HDR6A  4.200    2   180.00 ! analogy, BENZ
CD2R6L   CD2R6L   CD2R6A   HDR6A 4.200    2   180.00 ! analogy, BENZ

! aryl-aryl rotation dihedrals
CD2R6M   CD2R6L   CD2R6L   CD2R6M       0.3684  2   180.00 ! fitted
CD2R6A   CD2R6L   CD2R6L   CD2R6M       0.3685  2   180.00 ! fitted
CD2R6A   CD2R6L   CD2R6L   CD2R6A       0.3686  2   180.00 ! fitted
CD2R6M   OD30BN   PD1AN    OD30BN       0.3688  2     0.00 ! fitted
CD2R6A   CD2R6M   OD30BN   PD1AN        0.3687  2   180.00 ! fitted
CD2R6L   CD2R6M   OD30BN   PD1AN        0.3687  2   180.00 ! fitted
CD2R6M   OD30BN   PD1AN    OD2C2C       0.3686  2   180.00 ! fitted


! out-of-plane dihedrals
CD2R6L   CD2R6L   CD2R6M   CD2R6A       0.4897  2     0.00 ! fitted
CD2R6A   CD2R6A   CD2R6L   CD2R6L       1.5514  2   180.00 ! fitted

IMPROPERS
CD2R6L   CD2R6A   CD2R6L   CD2R6M       100.000   0     0.00
END
RETURN
```

References

[1] Tai-Sung Lee, Brian K. Radak, Anna Pabis, and Darrin M. York. A New Maximum Likelihood Approach for Free Energy Profile Construction from Molecular Simulations. *J. Chem. Theory Comput.*, 9(1):153–164, January 2013.

[2] Justin A. Lemkul, Jing Huang, Benoît Roux, and Alexander D. MacKerell. An Empirical Polarizable Force Field Based on the Classical Drude Oscillator Model: Development History and Recent Applications. *Chem. Rev.*, 116(9):4983–5013, May 2016.
